# Social isolation recruits amygdala-cortical circuitry to escalate alcohol drinking

**DOI:** 10.21203/rs.3.rs-4033115/v1

**Published:** 2024-03-21

**Authors:** Reesha R. Patel, Makenzie Patarino, Kelly Kim, Rachelle Pamintuan, Felix H. Taschbach, Hao Li, Christopher R. Lee, Aniek van Hoek, Rogelio Castro, Christian Cazares, Raymundo L. Miranda, Caroline Jia, Jeremy Delahanty, Kanha Batra, Laurel R. Keyes, Avraham Libster, Romy Wichmann, Talmo D. Pereira, Marcus K. Benna, Kay M. Tye

**Affiliations:** 1 Salk Institute for Biological Studies, La Jolla, CA, USA; 2 University of California San Diego, La Jolla, CA, USA; 3 Howard Hughes at Salk Institute, La Jolla, CA, USA; 4 Howard Hughes Investigator and Wylie Vale Professor at Salk Institute, La Jolla, CA, USA; 5 Kavli Institute for the Brain and Mind, La Jolla, CA, USA; 6 Center for Psychiatric Neuroscience, Northwestern University, Chicago, IL, USA

## Abstract

How do social factors impact the brain and contribute to increased alcohol drinking? We found that social rank predicts alcohol drinking, where subordinates drink more than dominants. Furthermore, social isolation escalates alcohol drinking, particularly impacting subordinates who display a greater increase in alcohol drinking compared to dominants. Using cellular resolution calcium imaging, we show that the basolateral amygdala-medial prefrontal cortex (BLA-mPFC) circuit predicts alcohol drinking in a rank-dependent manner, unlike non-specific BLA activity. The BLA-mPFC circuit becomes hyperexcitable during social isolation, detecting social isolation states. Mimicking the observed increases in BLA-mPFC activity using optogenetics was sufficient to increase alcohol drinking, suggesting the BLA-mPFC circuit may be a neural substrate for the negative impact of social isolation. To test the hypothesis that the BLA-mPFC circuit conveys a signal induced by social isolation to motivate alcohol consumption, we first determined if this circuit detects social information. Leveraging optogenetics in combination with calcium imaging and computer vision pose tracking, we found that BLA-mPFC circuitry governs social behavior and neural representation of social contact. We further show that BLA-mPFC stimulation mimics social isolation-induced mPFC encoding of sucrose and alcohol, and inhibition of the BLA-mPFC circuit decreases alcohol drinking following social isolation. Collectively, these data suggest the amygdala-cortical circuit mirrors a neural encoding state similar to social isolation and underlies social isolation-associated alcohol drinking.

## One Sentence Summary

The basolateral amygdala-to-medial prefrontal cortex circuit predicts alcohol drinking and is a key neural substrate of social isolation governing social contact and escalated alcohol drinking.

## INTRODUCTION

Social isolation can trigger the urge to drink alcohol, which became even more apparent in the aftermath of the recent COVID-19 pandemic. However, while alcohol drinking and sales went up during this time^1–3^, many individuals reduced or abstained from drinking^3^. This individual variability in responses to social isolation and alcohol drinking can be observed across many different vertebrate species, from humans to primates to rodents^4–7^. Isogenic mice that are housed under the same conditions show dramatic variability in their alcohol drinking^8,9^. Identifying mechanisms underlying individual variability in alcohol drinking could lead to preventative interventions of an alcohol use disorder in susceptible individuals. We hypothesized that social factors, including social rank and social isolation, could underlie such individual differences in alcohol drinking.

### Alcohol drinking correlates with social rank

**Fig. 1:**
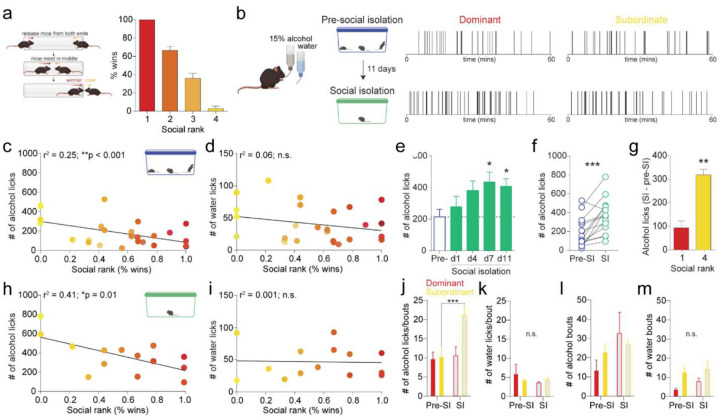
Social isolation increases alcohol drinking in a rank-dependent manner. **a,** Schematic of tube dominance task, and calculation of percent wins for each social rank by dividing the total number of trials won by the total number of trials received against any cage mate across 3 days of testing. **b,** Representative alcohol licking rasters from a dominant (rank 1; left) and subordinate (rank 4; right) mouse during two-bottle choice drinking before (top row) and during (bottom row) social isolation. **c,** Social rank is correlated with number of alcohol licks, where lower ranked mice showed more licking on the alcohol spout of a two-bottle choice (Pearson correlation, *r*^*2*^ = 0.25, **p < 0.01). **d,** No detectable correlation between social rank and number of water licks (Pearson correlation, *r*^*2*^ = 0.06, *p* = 0.21). **e,** Time course of escalated alcohol drinking during social isolation. **f,** Social isolation increased the number of alcohol licks (paired t-test, ***p < 0.001). **g,** Subordinate mice (rank 4) showed a larger escalation of alcohol licks following social isolation than Dominant mice (rank 1; unpaired t-test, **p < 0.01). **h,i,** Social rank is correlated with the number of alcohol licks during social isolation, where lower ranked mice showed more licking on the alcohol spout of a two-bottle choice (Pearson correlation, *r*^*2*^ = 0.41, **p < 0.01), but not for water (Pearson correlation, *r*^*2*^ = 0.002, *p* = 0.89) (I). **j,k,** Subordinate, but not Dominant, mice increase their alcohol drinking during social isolation by increasing the number of alcohol licks in each alcohol bout (*n* = 4 mice/group, two-way ANOVA, interaction effect: F (1, 6) = 20.84, ***p < 0.001 and main effect of social isolation: F (1, 6) = 28.84, **p < 0.01; Sidak’s post hoc, ***p < 0.001 compared to pre-SI; J), with no impact on water licks per bout (k). **l,m,** Social isolation increases alcohol drinking by increasing the number of alcohol bouts taken (two-way ANOVA, main effect of social isolation: F (1, 6) = 10.23, *p < 0.05; l), with no impact on the number of water bouts (**m**). Error bars indicate ±SEM.

To test this hypothesis, we first established the social rank of mice in each cage using the tube dominance test. In this task, mice engaged in a pairwise competition to push their cage mates out of the tube (Fig. 1a). Higher ranked, dominant mice outperformed their lower ranked, subordinate counterparts. Once stable social ranks were established, mice were given daily 1-hour access to two bottles containing 15% alcohol and water. We found a correlation between social rank with baseline alcohol, but not water, drinking (Fig. 1b–d; Supplementary Fig. 1 shows lick counts correlate with blood alcohol content), where subordinate mice drink more alcohol compared to dominant mice. These findings underscore the impact of pre-existing dominance hierarchies on behavioral adaptations in alcohol drinking.

### Social isolation increases alcohol drinking in a rank-dependent manner

Building on recent findings indicating social rank can influence responses to stress^10–12^, we sought to investigate how mice respond to a social isolation challenge, a condition with growing relevance to human experiences^13^. Social isolation increased drinking in all mice (Fig. 1e, f). Notably, lower ranked mice increase their drinking by a much greater magnitude than higher ranked mice during social isolation (Fig. 1g). Social rank remained strongly correlated with alcohol, but not water, drinking during social isolation (Fig. 1h, i). Interestingly, social isolation increased alcohol drinking through divergent behavioral mechanisms in dominant and subordinate mice, as reflected in their lick microstructure^14^. Subordinate mice increased their alcohol drinking by increasing the number of licks taken during each alcohol bout, while no effect was observed for water (Fig. 1j, k). Whereas dominant mice increased their alcohol drinking by increasing the number of alcohol bouts taken, no effect was observed for water (Fig. 1l, m). Together, these data suggest that social rank may predict individuals with a heightened propensity to intensify alcohol consumption during periods of social isolation.

### BLA-mPFC encodes and predicts alcohol drinking

The basolateral amygdala (BLA) plays a pivotal role in orchestrating emotional and stress responses^15,16^. More recently, the BLA has also been linked to social rank in humans and macaques^17,18^. These findings led us to investigate the potential involvement of BLA circuits in underlying the observed individual differences in alcohol drinking associated with social rank and social isolation. BLA neurons projecting to the medial prefrontal cortex (BLA-mPFC) were of particular interest given their role in social behavior^19^ and negative valence^19,20^, pointing toward a potential involvement in aversive social contexts such as social isolation.

**Fig. 2:**
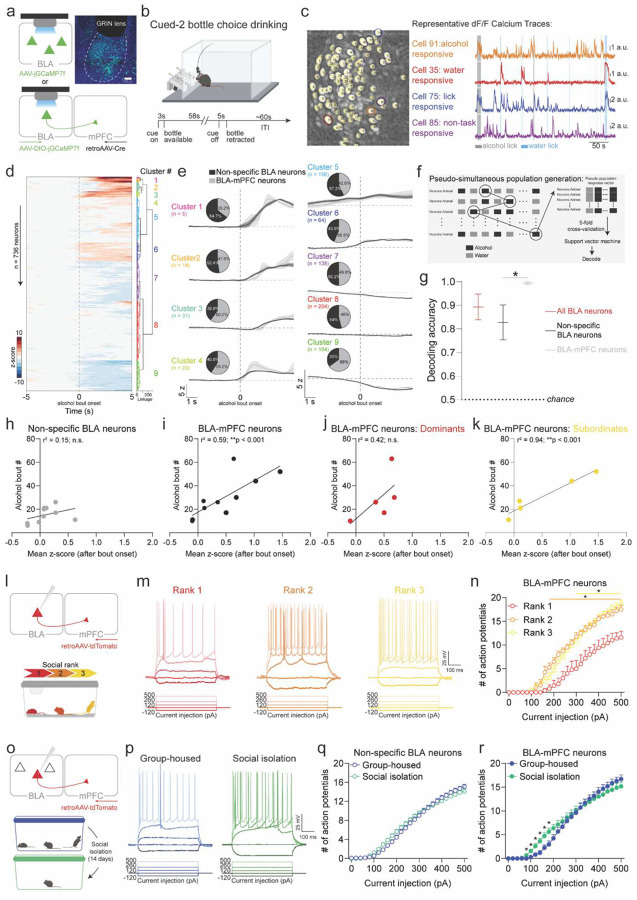
BLA-mPFC activity predicts alcohol drinking in a rank-dependent manner and is hyperexcitable following social isolation. **a,** Viral strategy and endoscopic lens implant for monitoring non-specific BLA and projection-specific BLA-mPFC neuronal activity. **b,** To record *in vivo* BLA dynamics during drinking, we designed a cued-two-bottle choice (cued-2BC) drinking task to enable conditioned availability of bottles and free-choice drinking, wherein a cue light signals availability of water and/or alcohol bottles for approximately a minute after which the bottles are retracted. **c,** Representative cell contour map and calcium traces showing heterogeneous responses to alcohol, water, and licking from BLA neurons recorded during cued-two-bottle choice drinking. **d,** Functional activity clusters of non-specific BLA and projection-specific BLA-mPFC neuronal responses to alcohol (Non-specific BLA: *n* = 540 neurons from *N* = 10 mice; BLA-mPFC projectors: *n* = 196 neurons from *N* = 11 mice). **e,** Projection-specific BLA-mPFC neurons showed enriched responses to alcohol compared to non-specific BLA neurons indicated in pie charts (insets) showing the proportion of non-specific BLA and BLA-mPFC neurons comprising each cluster, normalized by the number of neurons recorded from each group. **f,** Schematic of pseudo-simultaneous population sampling method used for decoding of alcohol versus water drinking from BLA population-level activity using a support vector machine. **g,** BLA-mPFC activity predicts alcohol drinking with the greatest accuracy compared to activity from all BLA or non-specific BLA neurons (one-way ANOVA; Kruskal-Wallis post hoc test, *p < 0.05). **h,** Non-specific BLA responses to alcohol did not correlate with the number of alcohol bouts (Pearson correlation, *r*^*2*^ = 0.15, *p* = 0.26). **i,** BLA-mPFC responses to alcohol predicts the number of alcohol bouts taken during cued-two-bottle choice drinking (Pearson correlation, *r*^*2*^ = 0.59, **p < 0.01). **j,** Higher ranked mice (ranks 1 and 2) showed no correlation between mean BLA-mPFC reponses to alcohol and the number of alcohol bouts taken during cued-2BC (Pearson correlation, *r*^*2*^ = 0.42, *p* = 0.23). **k,** Lower ranked mice (ranks 3 and 4) showed a significant correlation between mean BLA-mPFC responses to alcohol and the number of alcohol bouts taken during cued-two-bottle choice drinking (Pearson correlation, *r*^*2*^ = 0.94, **p < 0.01). **l,** Viral strategy to label and measure excitability of BLA-mPFC neurons using *ex vivo* patch-clamp electrophysiology across social rank. **m,** Representative action potential firing measured in BLA-mPFC neurons from Dominant (rank 1, red), Intermediate (rank 2, orange), and Subordinate (rank 3, yellow) mice using current-clamp recordings. **n,** Dominant mice showed reduced basal BLA-mPFC excitability compared to Intermediate and Subordinate mice (*n* = 4–12 neurons from *N* = 2–3 mice/group, two-way ANOVA, interaction effect: F (50, 250) = 5.52, ****p < 0.0001 and main effect of social rank: F (2, 18) = 6.49, **p < 0.01; Tukey’s post hoc, *p < 0.05 compared to rank 1). **o,** Viral strategy to label and measure excitability of BLA-mPFC neurons using *ex vivo* patch-clamp electrophysiology in group-housed and socially isolated mice. **p,** Representative action potential firing measured in BLA-mPFC neurons from group-housed (blue) and socially isolated (green) mice using current clamp recordings. **q,r,** Social isolation increases excitability of non-specific BLA neurons from all ranks (*n* = 80–76 neurons from *N* = 6–7 mice/group, two-way ANOVA, interaction effect: F (25, 3850) = 4.25, ****p < 0.0001; q) and BLA-mPFC neurons (*n* = 21–29 neurons from *N* = 5–6 mice/group, two-way ANOVA, interaction effect: F (25, 1200) = 4.45, ****p < 0.0001; Tukey’s post hoc, *p < 0.05; r). Error bars indicate ±SEM.

We first asked how BLA neurons respond to alcohol drinking. Using cellular resolution calcium imaging, we measured neural dynamics of non-specific BLA neurons and BLA-mPFC neurons while mice drank alcohol (Fig. 2a). We designed a novel drinking paradigm, cued two-bottle choice drinking (cued-2BC), that enabled both free-choice drinking as well as trial-structured, cued availability of bottles (Fig. 2b). During the cued-2BC task, a cue light indicated the availability of alcohol and water bottles, allowing the mice approximately a minute of access before the bottles are retracted and an intertrial interval period begins. We found diverse cellular responses, with neurons displaying increased activity in response to alcohol, water, both, as well as non-task related activity (Fig. 2c, d; Supplementary Fig. 2), indicating a heterogeneous neural representation in this task. Notably, non-specific BLA and BLA-mPFC neurons excited to alcohol and water consisted of largely non-overlapping ensembles (Supplementary Fig. 2). Hierarchical clustering of responses to alcohol for all recorded BLA neurons resulted in several functional clusters (Fig. 2d). Notably, a subset of alcohol-responsive functional clusters (clusters 3, 4, 6, 7, and 9) were enriched for BLA-mPFC neurons compared to non-specific BLA neurons (Fig. 2e), highlighting the involvement of BLA-mPFC circuits in alcohol responses. Using a support vector machine, we investigated whether population-level BLA activity is sufficient to decode alcohol versus water drinking (Fig. 2f). BLA-mPFC activity showed the highest decoding accuracy for alcohol drinking, compared to non-specific BLA neurons, suggesting greater alcohol-related information in BLA-mPFC neurons (Fig. 2g; Supplementary Fig. 3).

Mean BLA-mPFC responses to alcohol significantly correlated with the number of alcohol bouts taken, where greater BLA-mPFC activity predicts greater alcohol drinking, which was not observed in non-specific BLA neurons or water (Fig. 2h, i; Supplementary Fig. 4). Stratifying by social rank revealed a significant correlation specifically in subordinate, but not dominant, mice (Fig. 2j, k). Consistent with this, whole-cell patch-clamp recordings demonstrated social rank-dependent differences in basal BLA-mPFC excitability (Fig. 2l, m; Supplementary Table 2). Dominant mice showed significantly reduced excitability in this circuit (Fig. 2n), consistent with their lower basal drinking compared to subordinates. These data suggest that the BLA-mPFC circuit predicts alcohol drinking and reflects social-rank related differences.

### BLA-mPFC projectors are hyperexcitable following social isolation

To examine the impact of social isolation on the BLA-mPFC circuit, we used whole-cell patch-clamp electrophysiology and found that social isolation induced a more pronounced increase in BLA-mPFC excitability compared to non-specific BLA neurons (Fig. 2o–r; Supplementary Fig. 5, Supplementary Tables 1–3). To establish the causal role of the BLA-mPFC circuit in alcohol drinking, we mimicked the impact of social isolation using lick-triggered, closed-loop photoactivation of the BLA-mPFC circuit during 2-bottle alcohol and water choice drinking. Selective activation of BLA terminals in the mPFC increased alcohol, without affecting sucrose or water, drinking (Supplementary Fig. 6). Collectively, these findings suggest that social isolation may escalate alcohol drinking through modulation of the BLA-mPFC circuit.

### BLA-mPFC activity modulates social interaction following social isolation

**Fig. 3:**
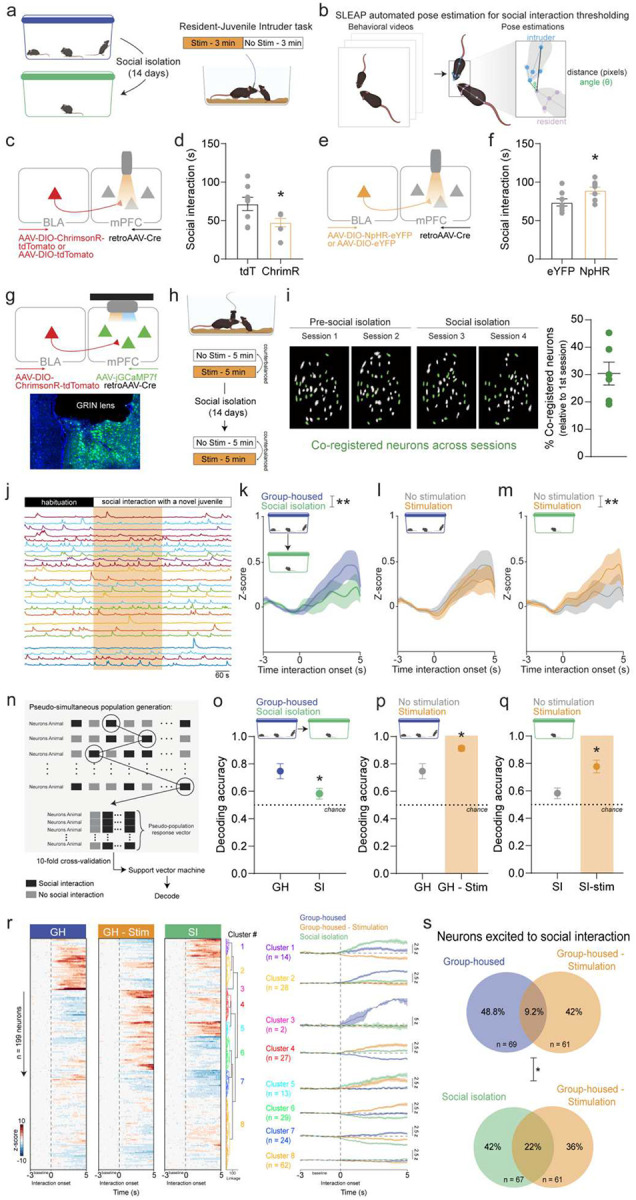
BLA-mPFC governs social behavior and neural representation of social contact **a,** Schematic of optogenetic stimulation epochs during resident-juvenile intruder task performed during social isolation. **b,** To precisely identify social interaction timeframes, SLEAP automated pose estimation was used to extract features, and a threshold of 60 pixels and 135 degrees for the distance and angle, respectively, between the resident’s head and intruder’s body was used to identify social interaction. **c,** Viral strategy used to activate BLA terminals in the mPFC during the resident-juvenile intruder task. **d,** Stimulation of BLA terminals in the mPFC decreased time spent socially interacting in the resident-juvenile intruder task during social isolation in ChrimsonR compared to tdTomato mice (unpaired t-test, *p < 0.05). **e,** Viral strategy used to inhibit BLA terminals in the mPFC during the resident-juvenile intruder task. **f,** Inhibition of BLA terminals in the mPFC increased time spent socially interacting in the resident-juvenile intruder task during social isolation in ChrimsonR compared to tdTomato mice (unpaired t-test, *p < 0.05). **g,** Viral strategy and endoscopic lens implant for simultaneous monitoring of mPFC activity and stimulation of BLA terminals in the mPFC. **h,** Calcium recordings during the resident-juvenile intruder task were performed before and during social isolation and BLA-mPFC stimulation was counterbalanced across two days. **i,** Representative cell contour maps showing an average of 30% of mPFC neurons were co-registered across all four sessions from before and during social isolation. **j,** Representative calcium traces during a BLA-mPFC stimulation session of the resident-juvenile intruder task. **k,** Social isolation decreases mean mPFC responses to social interaction (*n* = 199 neurons from *N* = 6 mice, two-way ANOVA, interaction effect: F (49, 19404) = 1.538, **p = 0.009). **l,** No detectable effect of BLA-mPFC terminal stimulation on mPFC population-level responses to social interaction bout onset before social isolation. **m,** BLA-mPFC terminal stimulation increases mean mPFC responses to social interaction during social isolation (two-way ANOVA, interaction effect: F (49, 7350) = 1.631, **p = 0.003). **n,** Schematic of pseudo-simultaneous population sampling method used for decoding social interaction from mPFC population-level activity using a support vector machine. **o,** Social isolation decreases decoding accuracy of mPFC activity in predicting social interaction behavior (unpaired t-test, *p < 0.05). **p,** BLA-mPFC terminal stimulation increases decoding accuracy of mPFC population-level activity in predicting social interaction behavior under pre-social isolation conditions (unpaired t-test, *p < 0.05). **q,** BLA-mPFC terminal stimulation increases decoding accuracy of mPFC population-level activity in predicting social interaction behavior under social isolation conditions (unpaired t-test, *p < 0.05). **r,** Functional activity clusters of mPFC neuronal responses to social interaction for pre-social isolation – no stimulation, pre-social isolation – stimulation, and social isolation- no stimulation sessions (*n* = 199 co-registered neurons from *N* = 6 mice). **s,** BLA-mPFC terminal stimulation recruited a greater proportion of the neurons excited to social interaction, as determined by Wilcoxon signed-rank test, during social isolation versus pre-social isolation conditions (*Chi*^*2*^ = 6.94, *p < 0.05). Error bars indicate ±SEM.

Despite the predictive power of BLA-mPFC activity, the precise mechanism by which this circuit influences alcohol drinking remained unclear. We hypothesized this circuit may convey a negative affective or ‘loneliness-like’ signal, which can motivate alcohol consumption^13,21^. It would then follow that the BLA-mPFC circuit detects social information. To determine this, we first asked if the BLA-mPFC circuit modulates social behavior. Using optogenetics, we manipulated this circuit during a resident-intruder task performed before and during social isolation and used SLEAP^22^ automated pose tracking to quantify social interaction (Fig. 3a, b; Supplementary Fig. 7). Stimulation of the BLA-mPFC reduced social interaction before and during social isolation (Fig. 3c, d; Supplementary Fig. 7). While BLA-mPFC inhibition increased social interaction selectively during social isolation (Fig. 3e, f). These data indicate a causal role of the BLA-mPFC circuit in promoting anti-social behavior seen following social isolation^23,24^.

### BLA-mPFC recruits a ‘social isolation ensemble’ to encode social contact

Given the observed role of the BLA-mPFC circuit in anti-social behavior, we next asked how the BLA influences mPFC encoding of social stimuli. To measure this, we used optogenetics in combination with cellular resolution calcium imaging, enabling us to capture mPFC dynamics while simultaneously stimulating BLA-mPFC terminals during resident-intruder interactions. We counterbalanced stimulated and non-stimulated resident-intruder sessions across two consecutive days, which was repeated after 14 days of social isolation (Fig. 3g, h). To determine how individual neural responses to social stimuli are shaped by social isolation experience, we tracked the same neurons across all resident-intruder sessions, resulting in an average co-registration of 30% of neurons across all four sessions (Fig. 3i). Representative calcium traces from a stimulation session can be seen in Fig. 3j. 14 days of social isolation reduced population-level mPFC responses to social interaction (Fig. 3k). In group-housed mice, BLA-mPFC terminal stimulation did not alter population-level mPFC responses to social interaction (Fig. 3l). However, following social isolation, BLA-mPFC terminal stimulation rescued social isolation-induced decreases in mPFC responses to social interaction (Fig. 3m). In line with this, using a support vector machine, mPFC population dynamics were sufficient to decode social interaction pre-social isolation, which was significantly reduced during social isolation (Fig. 3n, o; Supplementary Fig. 8). BLA-mPFC stimulation increased mPFC decoding of social interaction (Fig. 3p), suggesting that the BLA-mPFC circuit contributes to mPFC encoding of social behavior which ultimately leads to social avoidance. Notably, BLA-mPFC stimulation increased mPFC decoding of social interaction during social isolation (Fig. 3q). These data are consistent with the BLA-mPFC signaling heightened awareness of social stimuli, as typically seen following social isolation, to the cortex to enable anti-social behavior.

Individual mPFC neuronal responses to social interaction varied pre- and during social isolation as well as with BLA-mPFC stimulation as seen in the heatmap in which each row represents the same neuron’s response to social interaction under each condition (Fig. 3r). Hierarchical clustering revealed a stable functional cluster responding to social interaction pre-social isolation and during social isolation (cluster 1) as well as distinct functional clusters responding to social interaction under pre-social isolation (clusters 2 and 3) and during social isolation (cluster 5), suggesting that social isolation recruits distinct ensembles of neurons and potentially downstream circuits. Notably, one cluster (cluster 5) showed increased responses to social interaction during pre-social isolation BLA-mPFC stimulation and social isolation condition, suggesting that a similar ensemble of cells are recruited during pre-social isolation BLA-mPFC stimulation and social isolation. This led us to ask if the basal BLA-mPFC activation recruits more of a ‘social isolation ensemble’ to represent social interaction. To address this, we quantified the overlap in neurons excited to social interaction during pre-social isolation - no stimulation and pre-social isolation - BLA-mPFC stimulation conditions compared to the overlap in neurons excited to social interaction during social isolation - no stimulation and pre-social isolation - BLA-mPFC stimulation conditions. Notably, BLA-mPFC stimulation pre-social isolation led to recruitment of a greater proportion of ‘social isolation ensemble,’ that is neurons excited in response to social interaction, compared to the ‘pre-social isolation ensemble’ (Fig. 3s). No differences were observed in neurons inhibited to social interaction (Supplementary Fig. 9) These findings provide evidence that the BLA-mPFC circuit engages a neural ensemble encoding social interaction that resembles the neural signature of social isolation.

### BLA-mPFC stimulation mimics social isolation-induced mPFC alcohol encoding

**Fig. 4:**
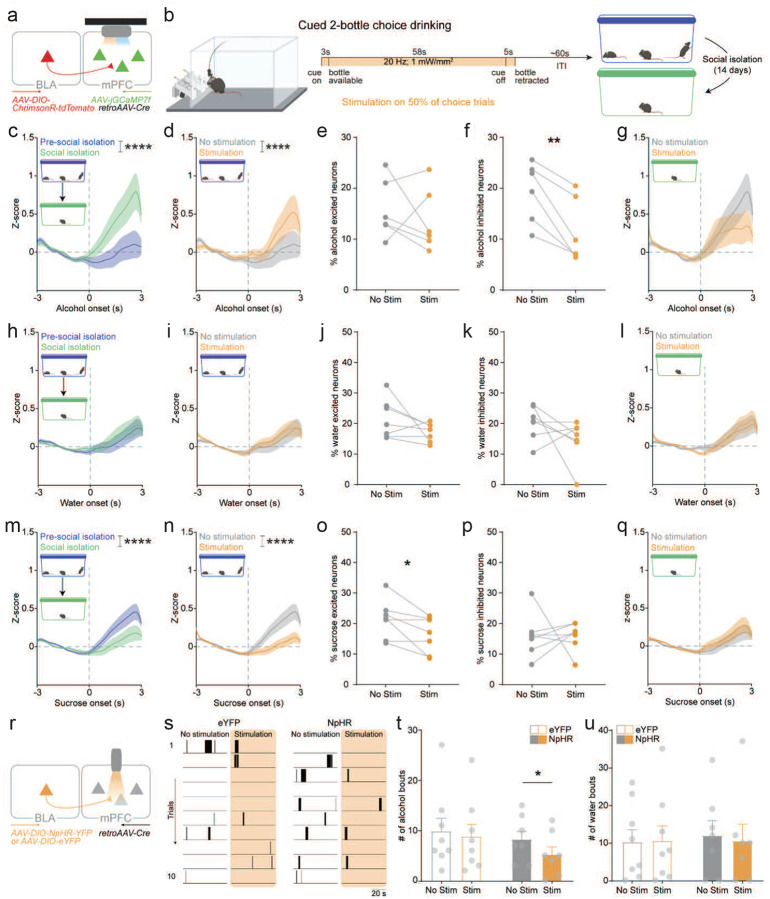
BLA-mPFC stimulation mimics social isolation-induced mPFC encoding of alcohol and sucrose and inhibition decreases alcohol drinking following social isolation. **a,** Viral strategy and endoscopic lens implant for simultaneous monitoring of mPFC activity and stimulation of BLA terminals in the mPFC. **b,** To measure the impact of BLA stimulation on mPFC dynamics during drinking, we used the cued-two-bottle choice drinking task, wherein BLA-mPFC stimulation occurred during 50% of trials, which was repeated pre- and during social isolation. **c,** Social isolation increased mPFC responses to alcohol drinking (*n* = 240–281 neurons from *N* = 5–6 mice, two-way ANOVA, interaction effect: F (60, 31659) = 2.198, ****p < 0.0001 and main effect of condition: F (1, 31659) = 101.9, ****p < 0.0001). **d,** BLA-mPFC stimulation increased mPFC responses to alcohol drinking under pre-social isolation conditions (*n* = 240 neurons from *N* = 6 mice, two-way ANOVA, main effect of stimulation: F (1, 29158) = 39.83, ****p < 0.0001), mimicking the effect of social isolation. **e,f,** BLA-mPFC stimulation decreased the proportion of mPFC neurons inhibited to alcohol (paired t-test, **p < 0.01; K) with no effect on mPFC neurons excited to alcohol. **g,** Social isolation occluded the effect of BLA-mPFC stimulation on mPFC responses to alcohol drinking (*n* = 281 neurons from *N* = 5 mice, two-way ANOVA, main effect of stimulation: F (1, 34160) = 5.352, **p = 0.002). **h-l,** No detectable effect of social isolation (M) or BLA-mPFC stimulation (N-Q) was observed for mPFC responses to water drinking. **m,** In contrast, social isolation decreased mPFC responses to sucrose drinking (*n* = 284–343 neurons from 7 mice, two-way ANOVA, main effect of condition: F (1, 19375) = 34.35, ****p < 0.0001). **n,** BLA-mPFC stimulation decreased mPFC responses to sucrose drinking under pre-social isolation conditions (*n* = 284 neurons from *N* = 7 mice, two-way ANOVA, interaction: F (60, 24526) = 2.982, ****p < 0.0001 and main effect of stimulation: F (1, 34526) = 103.2, ****p < 0.0001), mimicking the effect of social isolation. **o,p,** BLA-mPFC stimulation significantly decreased the proportion of mPFC neurons excited to sucrose (E) with no effect on mPFC neurons inhibited to sucrose (paired t-test, *p < 0.05) based on a mean Z-score response of ±1.98. **q,** Social isolation occluded the effect of BLA-mPFC stimulation on mPFC responses to sucrose drinking (*n* = 343 neurons from *N* = 7 mice). **r,** Viral strategy to inhibit BLA-mPFC activity during 50% of trials in the cued-two-bottle alcohol and water choice drinking task during social isolation. **s,** Representative licking raster during no stimulation and stimulation trials for eYFP (left) and NpHR (right) mice. **t,u,** BLA-mPFC inhibition decreased the number of alcohol bouts taken during stimulated versus unstimulated trials (*N* = 8 mice/group, two-way ANOVA, main effect of stimulation: F (1, 13) = 9.707, **p = 0.008); Sidak’s post hoc test, **p = 0.01; T) with no effect on water bouts taken (U). Error bars indicate ±SEM.

To further investigate how the BLA impacts mPFC encoding of alcohol, we again used a combined optogenetics and cellular resolution calcium imaging approach and measured mPFC dynamics while mice performed the cued-2BC drinking task before and following 14 days of social isolation. BLA-mPFC terminals were stimulated on 50% of trials in the cued-2BC drinking task (Fig. 4a, b). Social isolation increased mPFC responses to alcohol (Fig. 4c; Supplementary Fig. 10), which was also mimicked by BLA-mPFC stimulation (Fig. 4d; Supplementary Fig. 10). BLA-mPFC stimulation decreased the proportion of neurons inhibited in response to alcohol, without altering alcohol excited neurons (Fig. 4e, f). The effect of BLA-mPFC stimulation on mPFC responses to alcohol were occluded following social isolation (Fig. 4g; Supplementary Fig. 10 and 11), pointing to the involvement of this circuit by social isolation. Of note, no impact of social isolation or BLA-mPFC stimulation on mPFC responses to water were observed (Fig. 4h–l; Supplementary Fig. 10 and 11).

In contrast, social isolation decreased population-level mPFC responses to sucrose (Fig. 4m), suggesting a decrease in positive valence encoding in the mPFC. Remarkably, BLA-mPFC stimulation was sufficient to mimic the social isolation-induced decrease in mPFC responses to sucrose and significantly decreased the proportion of neurons excited by sucrose, without altering neurons inhibited by sucrose (Fig. 4n–p; Supplementary Fig. 10). The effect of BLA-mPFC stimulation on mPFC responses to sucrose were occluded following social isolation (Fig. 4q; Supplementary Fig. 10 and 11), highlighting engagement of this mechanism by social isolation. Together, these data suggests that BLA-mPFC circuit stimulation recapitulates a social isolation-like state of mPFC encoding.

### Inhibition of BLA-mPFC following social isolation reduces alcohol drinking

To test the causal role of the BLA-mPFC circuit in social isolation-associated drinking, we used optogenetics to inhibit the BLA-mPFC circuit in the cued-2BC task during social isolation. BLA-mPFC inhibition significantly decreased the number of alcohol bouts taken following social isolation, without altering water drinking (Fig. 4r–u), pointing toward a causal role of the BLA-mPFC circuit in social isolation-associated alcohol drinking. Taken together, these data suggest the amygdala-cortical circuit mimics a social isolation-like state of neural encoding and underlies behavioral adaptations including escalated alcohol drinking.

## Conclusion

We discovered that the BLA-mPFC circuit significantly influences alcohol consumption behaviors in response to social isolation, with effects modified by social rank. Subordinate mice display a heightened tendency for alcohol consumption compared to dominant ones. Using cellular resolution calcium imaging, we observed robust alcohol-related activity in the BLA-mPFC, predicting alcohol intake in a rank-dependent manner. Notably, social subordination is reflected in the basal excitability of the BLA-mPFC circuit, with social isolation inducing hyperexcitability in BLA-mPFC neurons. Optogenetic activation of this circuit mimics the heightened mPFC response to alcohol seen in socially isolated mice, suggesting a mechanism by which the BLA-mPFC circuit may drive alcohol consumption in isolated individuals. We investigated whether the BLA-mPFC circuit could induce a state similar to social isolation to drive alcohol consumption. We found that BLA-mPFC stimulation activates an ensemble mirroring the neural pattern associated with social isolation to encode social contact. Most notably, BLA-mPFC circuit stimulation mimics the heightened mPFC response to alcohol induced by social isolation. These findings highlight the BLA-mPFC circuit creates a state mirroring social isolation in mPFC encoding. Importantly, inhibiting the BLA-mPFC circuit post-isolation reduces alcohol intake, indicating a potential target for mitigating the negative effects of social isolation. These findings underscore the critical role of the BLA-mPFC circuit in mediating alcohol drinking behavior in response to social factors, shedding light on the neural mechanisms underlying susceptibility to alcohol use disorder.

## Supplementary Material

1

## Data Availability

All experimental data are available in the main text or supplementary material.
