# Supplementary material for: Social isolation recruits amygdala-cortical circuitry to escalate alcohol drinking
Source: Res Sq. 2024 Mar 21:rs.3.rs-4033115. Preprint. [Version 1] doi: 10.21203/rs.3.rs-4033115/v1 (PMC10984017; doi:10.21203/rs.3.rs-4033115/v1)
Supplement: 1 [file NIHPPrs4033115V1-supplement-1.pdf]

Methods and Materials

Animals and housing

Adult, male C57/BL6J mice (minimum of 10 weeks old) from Jackson Laboratory were used for all experiments described. Mice were housed 3-4 mice/cage on a 12-h reverse light/dark cycle with ad libitum access to food and water. All experiments were conducted during the dark cycle phase. A complete list of all mice used can be seen in **Supple. Table S4**. All experimental procedures were carried out in accordance with NIH guidelines and approval of the Salk Institutional Animal Care and Use Committee.

Stereotaxic surgeries

All surgeries were conducted under aseptic conditions. Briefly, mice were anesthetized with an isoflurane/oxygen mixture (4-5% for induction, 1-2% for maintenance) and placed in a stereotaxic head frame (David Kopf Instruments, Tujunga, CA, USA). A heating pad was placed under the mice to maintain body temperature, and Sterile Lubricant Eye Ointment (Stye, INSIGHT Pharmaceuticals Corp. Langhorne, PA) was applied to the eyes to prevent drying. The incision area was shaved and the skin was cleaned with alternating washes of 70% alcohol and betadine. A sub-cutaneous injection of lidocaine (0.5%) at the incision site for 3-5 minutes prior to surgery. An incision was made along the midline to expose the skull, and a dental drill was used to perform a craniotomy. During all surgeries, animals were injected subcutaneously with 1 mL of Ringer's solution, Buprenorphine (1 mg/kg), and Meloxicam (5 mg/kg). For recovery animals were placed in a clean cage on a heating pad. Animals were given >14 days for recovery before being subjected to behavioral paradigms.

All stereotaxic coordinates were measured relative to bregma and the top of the skull. Injections of viral vectors were performed using glass pipettes (Drummond Scientific) pulled to a 100-200 µm tip diameter with a pipette puller (Narishige PC-10, Amityville, NY, USA). Pipettes were either attached to 10 µL microsyringes (Hamilton Microlitre 701, Hamilton Co., Reno, NV, USA) with a microsyringe pump (UNP3; WPI, Worcester, MA, USA) and digital controller (Micro4; WPI, Worcester, MA, USA), or to the Nanoject III Programmable Nanoliter Injector (Drummond Scientific, Broomall, PA, USA) with digital controller (Drummond Scientific, Broomall, PA, USA). For each injection, micropipettes were slowly lowered to the target site and viral vectors were delivered at a rate of 1.0 nL per second. After 2 mins, the pipette was slowly raised 0.02 mm and left in place for another 8 minutes to allow diffusion of the virus before slowly being withdrawn.

To perform calcium imaging recordings, mice underwent surgery as described previously[8]. For non-specific BLA recordings, 200 nl of AAV1-Syn-jGCaMP7f-WPRE (Addgene), encoding GCaMP7f, was injected into the BLA (AP: -1.35 mm, ML: +3.4 mm , DV: −5.0 mm) and a 0.6 mm diameter by 7.3 mm length gradient refractive index lens with integrated baseplate (GRIN lens, Inscopix) was slowly lowered above the BLA (AP: -1.35 mm, ML: +3.4 mm , DV: −4.8 mm).

For projection-specific BLA-mPFC recordings, 200 nl of AAV1-Syn-FLEX-jGCaMP7f-WPRE (Addgene), cre-dependently encoding GCaMP7f was injected into the BLA (AP: -1.35 mm, ML: +3.4 mm , DV: −5.0 mm), 200 nl of retrogradely traveling AAV2-hSyn-Cre-P2A-tdTomato (Addgene), encoding Cre recombinase, was injected into the mPFC

(AP: +1.9 mm, ML: + 0.4 mm , DV: -1.9 mm), and a 0.6 mm diameter by 7.3 mm length GRIN lense with an integrated baseplate was slowly lowered above the ipsilateral BLA.

For simultaneous mPFC calcium recordings and BLA terminal stimulation, a mixture of 100 nL of AAV1-Syn-jGCaMP7f-WPRE and 100 nL of retrograde traveling AAV2-hSyn-Cre-P2A-tdTomato was injected into the mPFC (AP: +1.9 mm, ML: + 0.4 mm , DV: -1.9 mm), 200 nl of AAV8-hSyn-Flex-ChrimsonR-tdTomato (Addgene), encoding the ChrimsonR a red shifted excitatory opsin, was injected into the ipsilateral BLA (AP: -1.35 mm, ML: +3.4 mm , DV: −5.0 mm), and a 0.5 mm diameter by 4 mm length GRIN lens integrated with baseplate was implanted above the ipsilateral mPFC (AP: +1.9 mm, ML: + 0.4 mm , DV: -1.7 mm). No tissue was aspirated. All lens implants were secured to the skull with a thin layer of adhesive cement (C&B Metabond, Parkell), followed by black cranioplastic cement (Ortho-Jet, Lang). The implant was allowed to completely dry before closure of the incision with nylon sutures.

To label BLA-mPFC neurons for ex vivo electrophysiology, 200 nL of retrograde traveling AAV2-CAG-tdTomato, encoding a red fluorophore, was injected into mPFC (AP: +1.9 mm, ML: +0.4 mm , DV: -1.9 mm) of the right hemisphere.

For BLA-mPFC optogenetic excitation during the resident-intruder task, mice received unilateral injections of 200 nL of retrograde traveling AAV2-hSyn-Cre-P2A-tdTomato into the mPFC (AP: +1.9 mm, ML: + 0.4 mm , DV: -1.9 mm) and 200 nL of AAV8-hSyn-Flex-ChrimsonR-tdTomato or AAV8-Flex-tdTomato (Addgene) into the BLA (AP: -1.35 mm, ML: +3.4 mm , DV: −5.0 mm). A 300 μm diameter optic fiber (0.37 numerical aperture; RWD) encased in a 1.25 mm ferrule was then implanted with the fiber tip 0.30 mm above each injection site in the mPFC.

For BLA-mPFC optogenetic inhibition during the resident-intruder task and cued two-bottle choice drinking, mice received bilateral injections of 200 nL of retrograde traveling AAV2-hSyn-Cre-P2A-tdTomato into the mPFC (AP: +1.9 mm, ML: ±0.4 mm , DV: -1.9 mm) and 200 nL of AAV5-EF1-DIO-NpHR-eYFP or AAV5-EF1-DIO-eYFP (Addgene) into the BLA (AP: -1.35 mm, ML: ±3.4 mm , DV: −5.0 mm). A single 300 μm diameter optic fiber (0.37 numerical aperture) encased in a 1.25 mm ferrule was then implanted with the fiber tip 0.30 mm above the mPFC (AP: +1.9 mm, ML: + 0.0 mm, DV: -1.6 mm).

For BLA-mPFC optogenetic excitation during two-bottle choice drinking, mice received unilateral injections of 200 nL of AAV5-hSyn-ChR2-eYFP or AA5-hSyn-eYFP, encoding the excitatory channelrhodopsin or control yellow fluorophore respectively, into the BLA (AP: -1.35 mm, ML: +3.4 mm , DV: −5.0 mm). A 300 μm diameter optic fiber (0.37 numerical aperture) encased in a 1.25 mm ferrule was then implanted with the fiber tip 0.30 mm above the injection site in the mPFC.

All fiber implants were secured to the skull with a thin layer of adhesive cement followed by black cranioplastic cement. The implant was allowed to completely dry before closure of the incision with nylon sutures.

Behavioral assays

All behavioral testing occurred after a minimum of two weeks post-surgery recovery. Mice were handled for 15 minutes each day for three to five days to gain familiarity with experimenters and reduce stress during experiments.

Tube dominance test

The tube dominance test was used to assay the social rank of each mouse relative to their cage mates[25,26]. Mice were habituated to a small clear Plexiglas tube (7.5 cm length, 4.5 cm inner diameter) in their home cage for 1 to 3 days prior to tube training. Mice were individually trained to walk through a longer, clear Plexiglas tube (30 cm length, 3.2 cm inner diameter) over at least 3 days, until they were comfortably walking across without resistance. Following training, mice competed in 3 to 5 trials against each cagemate in a round-robin design and randomized order each day. For each trial, mice were released simultaneously into opposite ends of the tube, counterbalanced across trials, so that they met face-to-face in the center of the tube. The mouse which retreated from the confrontation was designated as the 'loser' and its opponent designated the 'winner'. An animal's 'relative social rank' was measured by the proportion of 'wins' across all contests from 3 to 4 days of testing. Social ranks obtained with the tube test were considered stable when obtaining the same results for 4 or more days in a row.

Two-bottle choice drinking

The two-bottle choice drinking (2BC) task was used to assay alcohol drinking before and during social isolation. Mice were given daily 1-hour sessions of 2BC in operant conditioning chambers (Med Associates Inc) equipped with white noise and two bottles that were wired for lick detection. To initiate drinking, a sucrose fade protocol was used, where water and an additional bottle of 10% sucrose-only was given until mice drank >500 licks on consecutive days. After this criteria was met, mice proceeded with the gradual sucrose fade in which bottles contained 10% sucrose + 15% (v/v) alcohol for days 1-3, 2% sucrose + 15% alcohol for days 4-6, and 15% alcohol-only for day 7 and onwards. Following 5-6 days of baseline alcohol 2BC sessions, mice were isolated for 11-14 days during which they received daily alcohol 2BC. The average number of alcohol licks was calculated for the last three days of baseline and for the 11 days of social isolation. Mice with less than 50 licks at the alcohol bottle were excluded from analysis for calcium recordings to ensure enough to ensure enough trials.

Calcium imaging during cued two-bottle choice drinking

To examine neural activity during alcohol drinking, we designed a hybrid behavioral paradigm that enabled both free-choice drinking as well as a trial structured, cued availability of bottles. For the cued two-bottle choice task (cued-2BC), operant conditioning chambers were equipped with white noise, two retractable bottles wired for lick detection (Med Associates Inc), and a cue light above each bottle. During cued-2BC, the cue light above the bottle was on for 3 seconds preceding availability of the bottle and remained on until 5 seconds before the bottle was retracted. The mice then had access to the bottles for 58 seconds after which the bottles were retracted and a random intertrial interval period ranging from 53 to 73 seconds began. Mice received a total of 30 trials consisting of 20 choice trials where both bottles were available, 5 forced water trials, and 5 forced alcohol/sucrose trials, where only the respective bottle was available.

The same 2BC drinking procedure as described above was used to initiate and maintain alcohol drinking with the following modifications. Following sucrose-only 2BC sessions,

mice were trained on the cued-2BC task with sucrose for 5 days and then the 2BC sucrose fade was conducted. After baseline alcohol 2BC, alcohol cued-2BC sessions were given. Calcium imaging recordings were obtained during the cued-2BC sessions. Mice were mounted with a miniscope or dummy miniscope for all sessions.

Optogenetic stimulation and calcium imaging during cued two-bottle choice drinking

For combined optogenetic and calcium imaging[27], the same cued two-bottle choice drinking procedure was used with the following modifications. An additional cued-2BC with stimulation was given after baseline cued-2BC sessions, in which mice received stimulation (20 Hz, 590-650 nm, 1 mW/mm^2) on 50% of choice trials in a random order. During stimulated trials, stimulation began at the onset of the cue and terminated with the bottles retracting. Following baseline testing, mice were isolated and given alcohol 2BC sessions. After 14 days of social isolation, mice were given reminder alcohol cued-2BC sessions without stimulation followed by an alcohol cued-2BC session with stimulation and repeated with sucrose cued-2BC sessions. Calcium imaging recordings were obtained during the cued-2BC stimulation sessions. Mice were mounted with a miniscope or dummy miniscope for all sessions and only received one session per day.

Optogenetic inhibition during cued two-bottle choice drinking

For optogenetic inhibition experiments, the same cued two-bottle choice drinking procedure was used as described above with the addition of a cued-2BC optogenetic inhibition session following the baseline cued-2BC and social isolation cued-2BC. Given the low drinking in this cohort of mice, we gave mice 2 weeks of the classic drinking in the dark (DID) paradigm[28] before the cued-2BC optogenetic inhibition session was conducted. DID consisted of daily 2-hour access to 15% (v/v) alcohol and water bottles in the home cage for four days followed by a 4-hour access on day 5 and two days with no access, which was repeated across 2 weeks. During stimulated trials in the cued-2BC session, continuous photoinhibition (589 nm, 15 mW at fiber tip) occurred at the onset of the cue and terminated with the bottles retracting during 50% of choice trials in a random order. Mice were tethered for all sessions.

Optogenetic stimulation during two-bottle choice drinking

For the photoactivation experiments during two-bottle choice drinking, we used a similar two-bottle choice drinking paradigm as described above. Following baseline, a test session was given, in which lick onset at either the alcohol or waterspout triggered 5 seconds of blue light delivery (20 Hz pulse, 479 nm, 9 mW at fiber tip). Mice were then given a recovery session where no photostimulation was delivered. After alcohol sessions, a baseline session of two-bottle choice drinking with 1% sucrose and water was given followed by a test session with optogenetic stimulation during sucrose drinking. Mice were tethered for all sessions.

Optogenetic stimulation during resident-juvenile intruder task

Mice were tested individually in their home cage. After a 3-minute habituation period where mice were able to freely explore alone, a novel juvenile mouse was placed in the cage for 6 minutes during which they received a stimulation epoch during the first 3 minutes followed by no stimulation for 3 minutes. The task was repeated after 14 days of social isolation with a different juvenile mouse. Stimulation parameters were as follows: ChrimsonR, 20 Hz, 589 nm, 15 mW at fiber tip and NpHR, continuous, 589 nm,

15 mW at fiber tip. The behavior of the mouse during the 3-minute stimulation epoch with the juvenile was used to calculate social interaction using SLEAP automated pose tracking (described below).

Optogenetic stimulation and calcium imaging during resident-juvenile intruder task

Mice were tested individually in their home cage. Mice were mounted with miniscopes (nVoke, Inscopix) and allowed a 5-minute habituation period where they were able to freely explore alone. After which, a novel juvenile mouse was placed in the cage for 5 minutes during which mice received stimulation (20 Hz, 590-650 nm, 1 mW/mm^2) or no stimulation, counterbalanced across two consecutive days. This procedure was repeated after 14 days of social isolation. A novel juvenile mouse was used for each session. The behavior of the mouse during the 5-minute interaction period with the juvenile was used to calculate social interaction using SLEAP automated pose tracking (described below).

Lick microstructure analysis

Microstructure analysis of lick contacts to bottles was conducted using custom MATLAB code. A bout was defined as two consecutive licks within 1 second of each other, with the onset of the bout being the first lick. The number of licks per bout and total number of bouts in the session were accordingly calculated. For calcium recordings only bouts separated by a minimum of 3 seconds (the period used for baseline calculation) were used for analysis.

Calcium imaging analysis

Calcium imaging data acquisition and calcium signal extraction

nVista and nVoke Inscopix systems were used to collect calcium imaging data. During behavior, a TTL signal was used to trigger the miniscope recording for the duration of the session. The miniscope was connected to an active commutator (Inscopix).

Image processing was accomplished using IDAS software (Inscopix). Raw videos were pre-processed by applying 4x spatial downsampling to reduce file size and processing time. A temporal downsampling was applied for a final frame rate of 10hz. Images were cropped to remove post-registration borders and sections in which cells were not observed. Motion was corrected for by using the first frame as a reference frame. Videos were then exported as TIFF stacks for analysis and converted to an 8-bit TIF in Fiji[29]. We used the constrained non-negative matrix factorization algorithm optimized for micro-endoscopic imaging (CNMF-E)[30] to extract fluorescence traces from neurons. Since cellular calcium fluctuations can exhibit negative transients associated decreases in firing, we did not apply non-negative constraints on temporal components[8]. All neurons were visually confirmed, and neurons exhibiting abnormalities in morphology and calcium trace were excluded. Neuron curation was performed by experimenters blinded to the experimental condition.

Population-level mean response calculation

To calculate the neuronal response to drinking and social interaction bouts, the GCaMP7f fluorescence signal for each neuron was Z-score normalized to a 3 or 5 second baseline period immediately preceding the onset of the bout. Z-scores were calculated as $(F(t) - Fm)/SD$ where $F(t)$ is the $\Delta F/F_0$ at time t and Fm is the mean of

$\Delta F/F_0$ in a baseline period. The Z-scored normalized trace was then averaged across a matched number of bouts for each condition for each neuron. The population mean response was then calculated by averaging the mean Z-scored normalized trace of all neurons. The proportion of responsive neurons was calculated using either the Wilcoxon signed-rank test or defined by a mean Z-score of ±1.98 calculated from bout onset to 3 seconds.

For calcium recordings during resident-juvenile intruder, the same neurons were co-registered across all social interaction sessions using the cell registration algorithm developed by Sheintuch *et al.*[31] through an open sourced MATLAB based GUI, CellReg. Briefly, spatial footprints of neurons, from CNMF-E output, for all sessions were aligned to a reference session through rotational and translational shifts. Cell pairs were identified by employing a Bayesian probability method that considers the centroid distance between cells and their spatial correlation.

Agglomerative hierarchical clustering

Each neuron's mean Z-score trace was used for agglomerative hierarchical clustering with a Ward linkage method and Euclidean distance metric. The threshold cut-off for maximum linkage was set to the highest cutoff that resulted in at least one cluster that showed no response. All neurons from each cluster were then averaged to create a peri-event time histogram of activity for each cluster. For co-registered neurons, each neuron's mean Z-score response before and during social isolation were first horizontally concatenated using a custom MATLAB script prior to agglomerative hierarchical clustering. All neurons from each cluster were then averaged to create a peri-event time histogram of activity for each cluster before and during social isolation.

Pseudo-simultaneous population generation and decoding for alcohol drinking

In each experimental session and for every individual, drinking bouts lasting more than 1s were identified, with each bout being distinct and separated by a minimum interval of 2 seconds from another. Bouts were categorized based on whether the individual was consuming alcohol (or sucrose) or water, thereby defining our two conditions.

Following the identification, we performed a k-fold cross-validation procedure. This procedure was executed ensuring that each fold contained at least one bout from each class. By ensuring this representation in every fold, we aimed to guarantee an unbiased and comprehensive evaluation of our machine learning models.

Subsequently, we generated pseudo-simultaneous training and testing populations from the respective datasets of all subjects[17,32]. In this context, a pseudo-simultaneous population refers to an aggregated or amalgamated set of neuronal activity data gathered from multiple animals. The purpose of this construction is to simulate a larger, collective neuronal population, enabling more robust computational or statistical analysis.

The process of creating this pseudo-simultaneous population involved several steps. First, for each subject, we pooled neuronal activity data corresponding to the identified bouts of drinking behavior. Next, we sampled a behavioral label (0 or 1) and pseudo-randomly sampled a single timepoint from each subject corresponding to that behavioral label. The neural activity of all animals can then be stacked creating a single vector of shape 1 x N, where N is the total number of neurons across all animals. This was then

repeated T times (T=10,000 unless otherwise noted) to create a pseudo-simultaneous population of shape T x N. We then followed the same procedure to create a testing population using the testing data.

The resultant training and testing pseudo-simultaneous populations were then used to train and evaluate our linear Support Vector Machine classifiers. The entire procedure was repeated for 'k' iterations, as defined in the k-fold cross-validation scheme. The training data was balanced by subsampling the majority-class before training the model to avoid introducing bias.

Creating the pseudo-simultaneous population for the social interaction data was identical to the method outlined above. However, instead of separating the data into drinking bouts, it was separated into bouts of interaction, and bouts of noninteraction. Noninteraction bouts that were longer than 20 seconds were divided into multiple smaller bouts while still separating each bout by at least 2 seconds.

SLEAP automated pose tracking analysis

To automatically detect mouse social interaction behavior, Social LEAP Estimates Animal Poses (SLEAP)[22], was used to estimate animal poses in behavioral videos. Behavioral videos were attained using Noldus EthoVision XT and a Basler GenICam at 25 frames/second. A training data set was labeled using a 6-point skeleton to represent the mouse (nose, left ear, right ear, skull base, haunch, and tail base). This data set was used to train bottom-up models with 798 labeled frame for each model for Chrimson and NpHR, in which the location of each labeled body part is identified and then assigned to the appropriate mouse. All frames were visually confirmed for correct labeling of identity. To identify social interaction frames for optogenetic experiments, a threshold of 60 pixels for distance and 135-degree angle between the resident's head and intruder's body was used to identify social interaction. Note, cameras were positioned a fixed distance above the cage for all recordings. These thresholds were validated with manually scored data and are consistent with findings from unsupervised clustering methods described below (**Supplementary Fig. 7**).

To identify social interaction frames for decoding behavior from calcium data, we calculated features, from the SLEAP-predicted coordinates of labeled body points, describing the relative locations between the resident mouse and intruder mouse, including head-to-head distance and angle, resident's head-to-intruder's tail base distance and angle, intruder's head-to-resident's tail base distance and angle, distance between the resident and intruder's tail base and resident's velocity, and intruder's velocity. To identify distinct behavioral motifs mice exhibited during social interaction, we applied Uniform Manifold Approximation and Projection (UMAP) onto these behavioral features and obtained unsupervised clusters. Clusters were manually annotated as social or non-social clusters based on visualization of each cluster by generating a video from the frames included in that cluster.

Ex vivo whole-cell patch-clamp electrophysiology

A separate cohort of mice were injected as described above in '*Stereotaxic surgeries'* to label BLA-mPFC projection neurons for whole-cell patch-clamp recordings. Following 4 weeks of postoperative recovery, mice underwent tube dominance testing to assess the social rank of each mouse in the cage. After stable social ranks were established, mice

were isolated for 14-16 days or remained group-housed before patch-clamp recordings were obtained.

Mice were anesthetized with sodium pentobarbital (200 mg/kg-1; intraperitoneal injection), decapitated, and brains were quickly extracted in oxygenated (95% O2 and 5% CO2) high sucrose cutting solution containing (in mM): 87 NaCl, 2.5 KCl, 1.3 NaH2PO4, 7 MgCl2, 25 NHCO3, 75 sucrose, 5 ascorbate, and 0.5 CaCl2 (~320-300 mOsm; pH = 7.3). Coronal sections (300 µm) containing the mPFC and BLA were collected using a vibrating blade microtome (VT120; Leica, Buffalo Grove, IL). Slices were then incubated in oxygenated, modified artificial cerebrospinal fluid (ACSF) containing (in mM): 75 sucrose, 87 NaCL, 2.5 KCl, 1.3 NaH2PO4, 7 MgCl2, 0.5 CaCl2, 25 NaHCO3 and 5 ascorbic acid (~300 mOsm; pH = 7.3) in a 31°C water bath for 1 hour before recordings were obtained. Viral injections sites in the mPFC were visually confirmed.

Slices were visualized through an upright microscope (Scientifica, UK) equipped with infrared-differential interference contrast (IR-DIC) optics and a Q-imaging Retiga Exi camera (Q Imaging, Canada). Whole-cell patch-clamp recordings were obtained from visually identified neurons expressing tdTomato and adjacent, neighboring non-expressing neurons. Neuronal recordings were obtained using glass pipettes with a pipette resistance of 3-5 MΩ fabricated with a horizontal puller (P-1000, Sutter, CA) and filled with internal solution containing (in mM): 125 potassium gluconate, 20 HEPES, 10 NaCl, 3 MgATP, and 8 biocytin (~290 mOsm; pH = 7.3). Recorded signals were acquired, amplified, and digitized at 10kHz using a MultiClamp 700B amplifier, Digidata144, and pClamp10 software (Molecular Devices, Sunnyvale, CA). During recordings, the brain slice was maintained in a bath with continuously perfused oxygenated ACSF at 31 ±1°C using a peristaltic pump (Minipuls3; Gilson, WI, USA).

To examine the membrane potential response to current injection, neurons were voltage-clamped at -70 mV and given a series of 500 ms current injections ranging from -120 to 500 pA in 20 pA increments. Data were analyzed using Clampfit software (Molecular Devices, Sunnyvale, CA) and GraphPad Prism (GraphPad Prism, La Jolla, CA). The number of action potentials elicited by increasing current injections was averaged across neurons and plotted to generate excitability curves. The minimum current required to elicit action potential firing was recorded as the threshold. Membrane properties including cell capacitance and resting membrane potential were recorded or input resistance estimated from the steady-state membrane response to a -120 pA hyperpolarizing current injection. Images depicting the location of the recording pipette within the slice were captured through a 4X/0.10 NA objective. Images were subsequently overlaid onto the appropriate brain atlas image and recorded cell locations were annotated (as seen in **Supplementary Fig. 4**).

Blood alcohol concentration

Blood alcohol concentration was assessed to determine if measurements of alcohol licking correlated with alcohol consumption. Following experimental testing, terminal trunk blood was collected from mice immediately after completion of a two-bottle choice drinking session. Blood was collected in an Eppendorf tube and immediately centrifuged to separate serum. Blood alcohol content was measured on an Agilent 7820A GC

coupled to a 7697A (headspace-flame-ionization) through the Scripps Research Animal Models Core.

Histology

Following experiments, mice were deeply anesthetized with sodium pentobarbital (200 mg/kg, intraperitoneal injection). Animals were transcardially perfused with 10 mL of Ringer's solution followed by 10 mL of cold 4% PFA in 1X PBS. For viral injection verification, mice were immediately decapitated, brains were extracted and fixed in 4% PFA in 1X PBS for 24 hours at 4°C. For optic fiber and GRIN lens verification, mice were immediately decapitated and the whole head was submerged in 4% PFA in 1X PBS for 24 hours at 4°C. The following day the fiber optic implants/GRIN lens were removed, and the brain was extracted. Brains were then transferred to 30% sucrose in 1X PBS at 4°C until they sunk to the bottom of the sucrose solution. Brains were sectioned coronally at 50µm using a microtome (ThermoScientific and were stored at 4°C in 1X PBS until staining. Sections were mounted directly onto glass microscope slides and cover slipped with EMS-Shield Mounting Medium w/ DAPI. Slides were then imaged at 2x using the Keyence BZX710 Fluorescence microscope. Injection sites and implants relative to the mouse atlas were annotated (**Supplementary Fig. 12**).

Statistics

Statistical analyses were performed using GraphPad Prism (GraphPad Prism, La Jolla, CA) and MATLAB (Mathworks, Natick, MA). Data with a Gaussian distribution were compared using a paired or unpaired t-test (non-directional) for two experimental groups, and a one-way or two-way ANOVA with repeated measures for three or more experimental groups. Correlation between two variables was assessed using the Pearson's correlation coefficient. Chi-square statistics were used to detect differences in distribution between two groups. Threshold for significance was placed at $p < 0.05$. All data are shown as mean ± standard error of the mean (SEM).

a

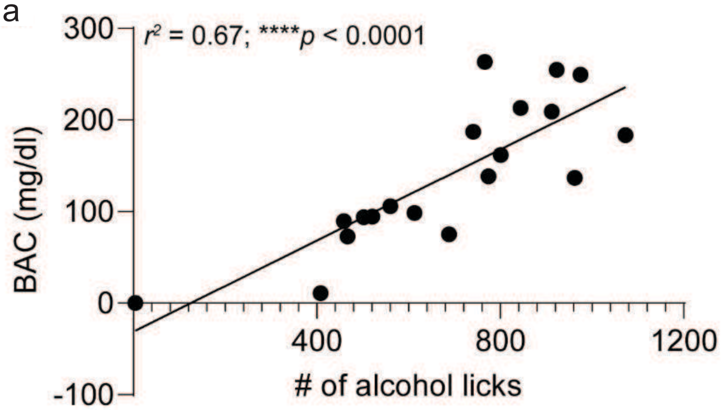

Supplementary Figure 1. Alcohol licking is correlated with blood alcohol levels.

a, The number of licks detected at the alcohol bottle wired to a lickometer correlated with blood alcohol concentrations, demonstrating that these are reliable measures of alcohol consumption (Pearson's correlation, $r^2 = 0.67$, ****$p < 0.0001$).

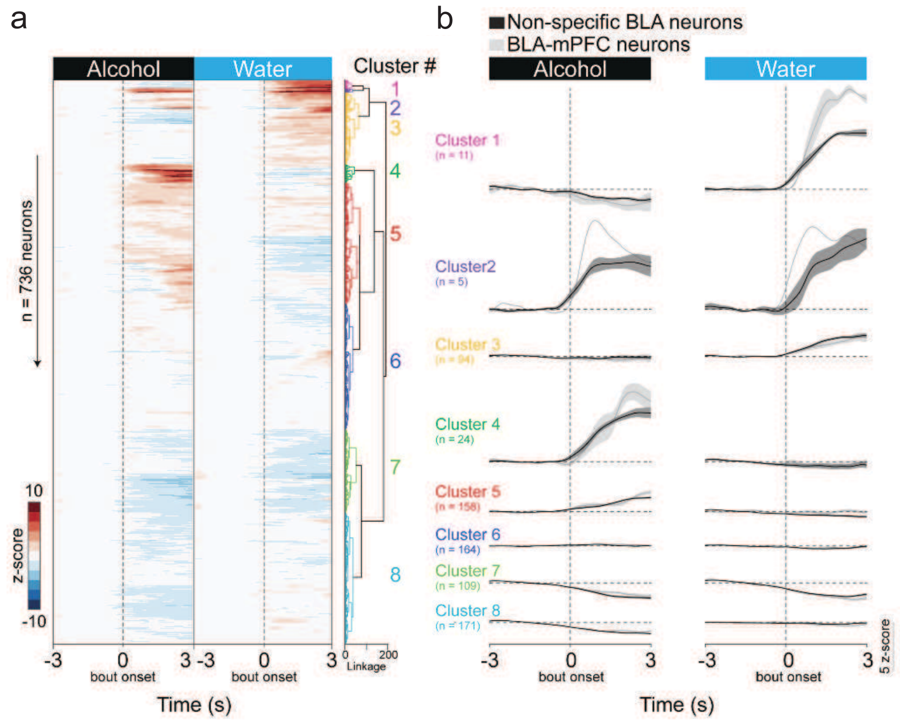

Supplementary Figure 2. Non-specific BLA and BLA-mPFC neurons excited to alcohol and water consisted of largely non-overlapping ensembles.

a, Functional activity clusters of combined non-specific BLA and BLA-mPFC neuronal responses to alcohol and water (*n* = 736 neurons from *N* = 21 mice). **b,** Mean responses of each functional cluster stratified by non-specific BLA and BLA-mPFC neuronal populations.

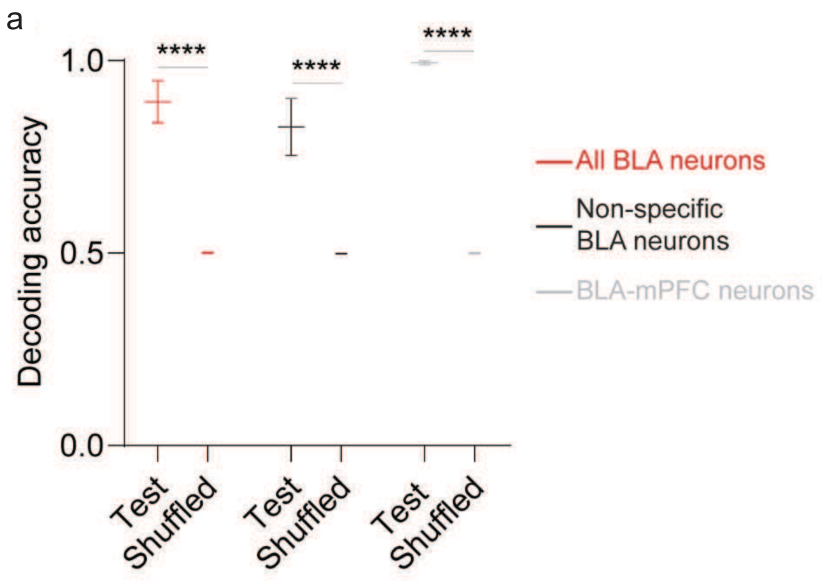

Supplementary Figure 3. BLA activity significantly decodes alcohol drinking.

a, Population-level non-specific BLA neuron, BLA-mPFC neuron, or the combined all BLA neuron activity decodes alcohol versus water drinking compared to chance levels as determined by shuffled data using a support vector machine (two-way ANOVA, main effect: $F(1, 24) = 174.2$, **** $p < 0.0001$; Sidak's post hoc test, ****$p < 0.0001$). Error bars indicate ±SEM.

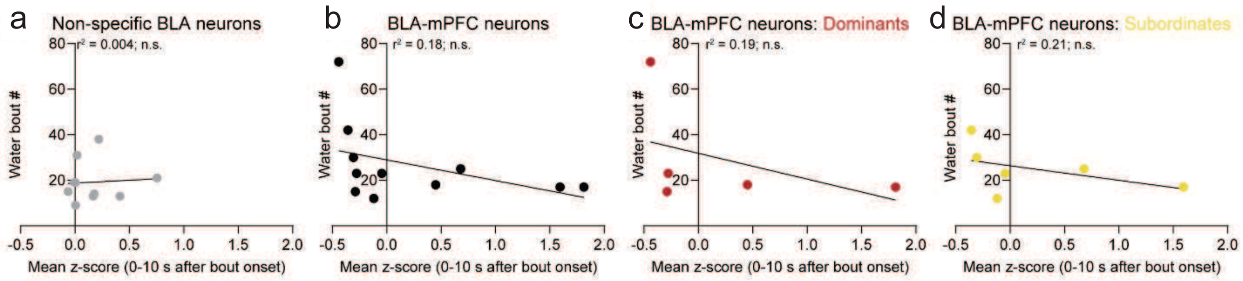

Supplementary Figure 4. BLA activity does not correlate with water drinking.

a,b, Non-specific BLA (Pearson correlation, $r^2 = 0.004$, $p = 0.84$; a) and BLA-mPFC (Pearson correlation, $r^2 = 0.17$, $p = 0.19$; b) activity was not correlated with water drinking. **c,d,** No correlation was observed between BLA-mPFC activity and water drinking in dominant (Pearson correlation, $r^2 = 0.19$, $p = 0.45$; c) or subordinate (Pearson correlation, $r^2 = 0.21$, $p = 0.35$; d) mice.

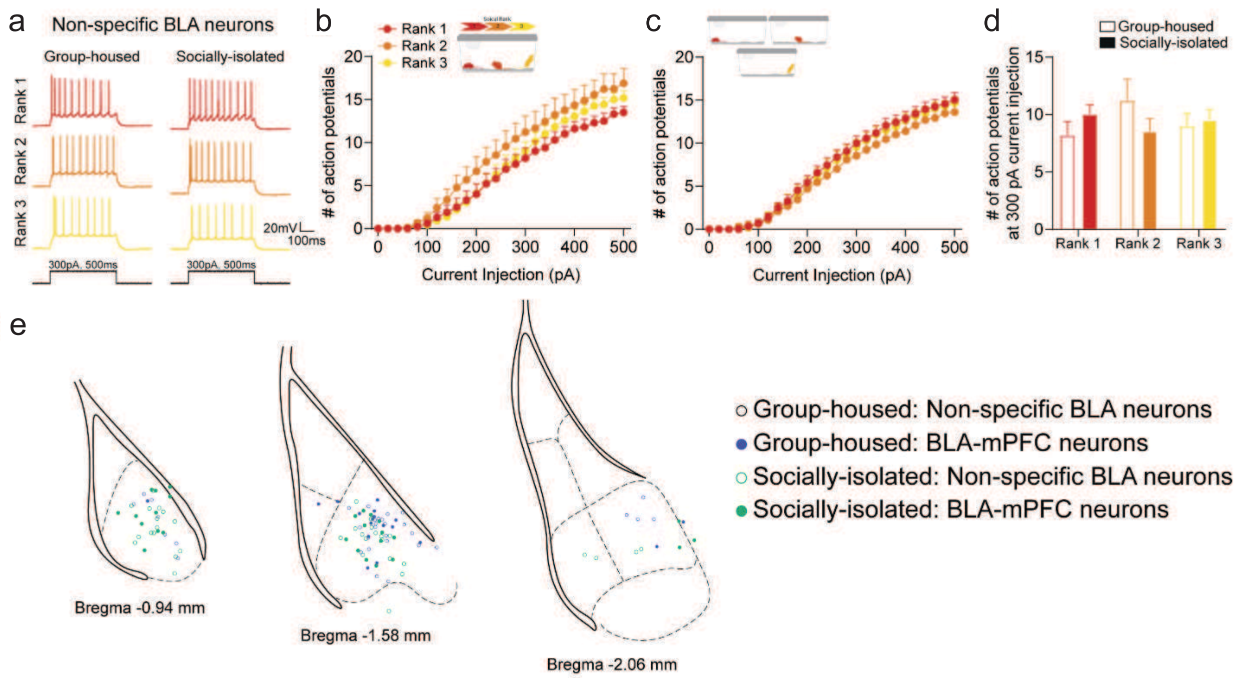

Supplementary Figure 5. Non-specific BLA neurons showed no social rank-dependent differences.

a, Representative action potential firing measured in non-specific BLA neurons from group-housed and socially isolated mice for each social rank. **b,c,** No detectable social rank-dependent differences were observed in non-specific BLA neurons from group-housed (n = 9-16 cells from N = 3 mice/rank; b) or socially isolated mice (n = 11-16 cells from N = 3 mice/rank; c). **d,** Social isolation did not alter the number of action potentials elicited by a 300pA current injection across each social rank. **e,** Anatomical location of recorded BLA neurons. Error bars indicate ±SEM.

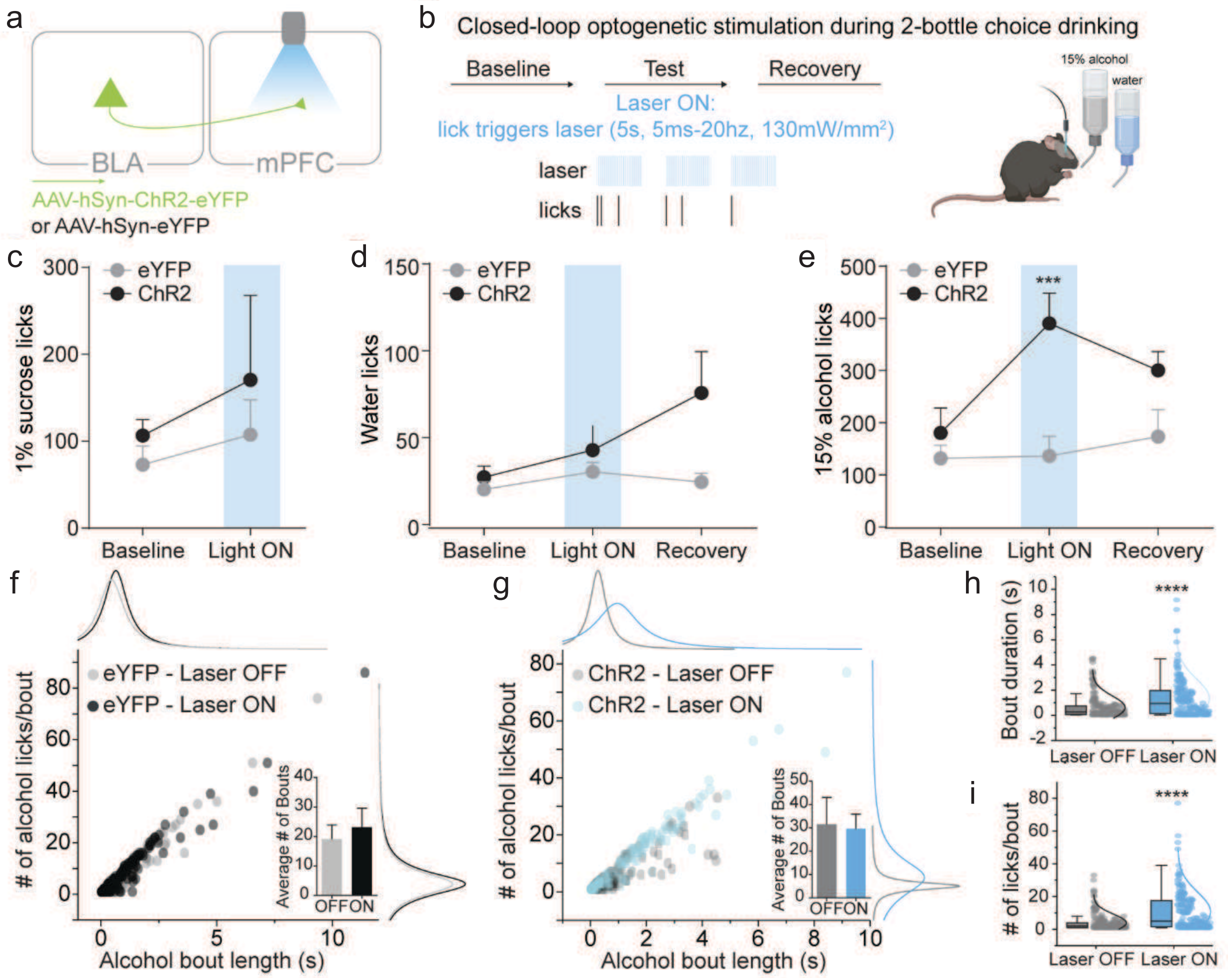

Supplementary Figure 6. BLA-mPFC terminal stimulation increases alcohol drinking

a, Viral strategy to activate BLA terminals in the mPFC during two-bottle choice drinking. **b,** To determine how BLA-mPFC circuits impacts alcohol drinking, we used lick-triggered closed-loop optogenetics during two-bottle alcohol and water choice drinking, where licking at either bottle resulted in a 5 second stimulation. **c,d,** BLA-mPFC stimulation did not alter sucrose (c) or water (d) drinking. **e,** BLA-mPFC stimulation increased alcohol drinking (N = 6 mice/group, two-way ANOVA). **f,g,** Distribution of alcohol bout lengths and number of licks per bout during non-stimulated and BLA-mPFC stimulation conditions for eYFP control (f) and ChR2 (g) mice. **h,i** BLA-mPFC stimulation increased alcohol bout duration (unpaired t-test, ****$p < 0.0001$; h) and the number of alcohol licks per bout (unpaired t-test, ****$p < 0.0001$; i). Error bars indicate ±SEM.

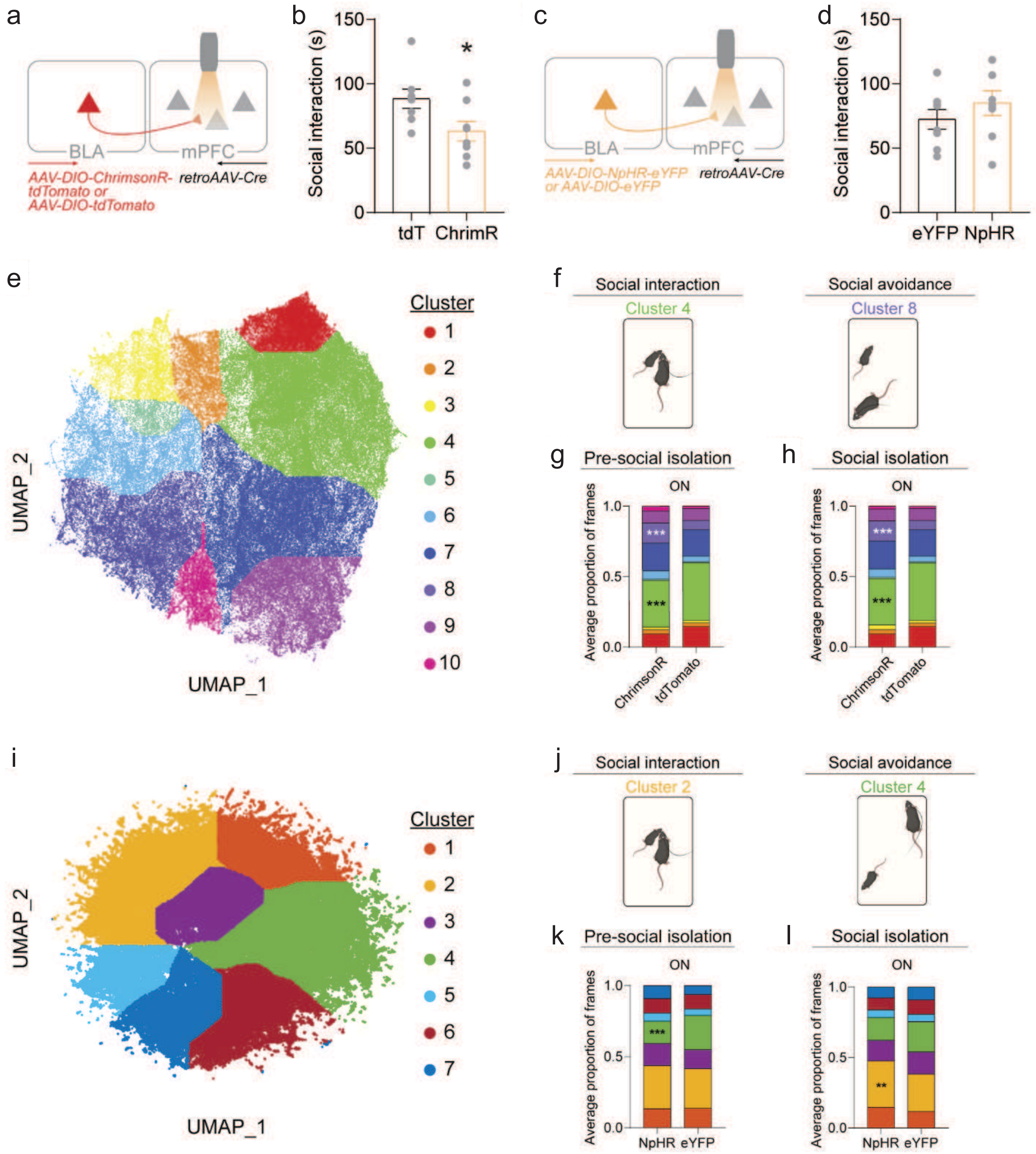

Supplementary Figure 7. BLA-mPFC activation induced anti-social behavior.

a, Viral strategy used to activate BLA terminals in the mPFC during the resident-juvenile intruder task. **b,** Stimulation of BLA terminals in the mPFC decreased time spent socially interacting in the resident-juvenile intruder task under group-housed conditions (unpaired t-test, $*p < 0.05$). **c,** Viral strategy used to inhibit BLA terminals in the mPFC during the resident-juvenile intruder task. **d,** Inhibition of BLA terminals in the mPFC did not alter time spent socially interacting in the resident-juvenile intruder task under

group-housed conditions. **e,** Representative unsupervised UMAP clustering of all behavioral frames from all ChrimsonR and tdTomato mice. **f,** Schematic depicting a sample frame from identified social interaction and social avoidance behavioral clusters. **g,** Average proportion of frames in each cluster for ChrimsonR and tdTomato mice during the pre-social isolation stimulation epoch showing a significant decrease in social interaction in ChrimsonR mice (two-way ANOVA, interaction effect: $F(9,140) = 4.94$, ****$p < 0 .0001$, Sidak's post hoc test, ***$p < 0.001$, $N = 8$ mice/group). **h,** Average proportion of frames in each cluster for ChrimsonR and tdTomato mice during the social isolation stimulation epoch showing a significant decrease in social interaction in ChrimsonR mice (two-way ANOVA, interaction effect: $F(9,140) = 4.99$, ****$p < 0 .0001$, Sidak's post hoc test, ***$p < 0.001$, $N = 8$ mice/group). **I,** Representative unsupervised UMAP clustering of all behavioral frames from all NpHR and eYFP mice. **j,** Schematic depicting a sample frame from identified social interaction and social avoidance behavioral clusters of significant differences. **k,** Average proportion of frames in each cluster for NpHR and eYFP mice during the pre-social isolation stimulation epoch showing a significant decrease in social avoidance in NpHR mice (two-way ANOVA, interaction effect: $F(6,98) = 4.02$, **$p < 0 .01$, Sidak's post hoc test, ***$p < 0.001$, $N = 8$ mice/group). **l,** Average proportion of frames in each cluster for NpHR and eYFP mice during the social isolation stimulation epoch showing a significant increase in social interaction in NpHR mice (two-way ANOVA, interaction effect: $F(6,98) = 3.63$, **$p < 0 .01$, Sidak's post hoc test, **$p < 0.01$, $N = 8$ mice/group). Error bars indicate ±SEM.

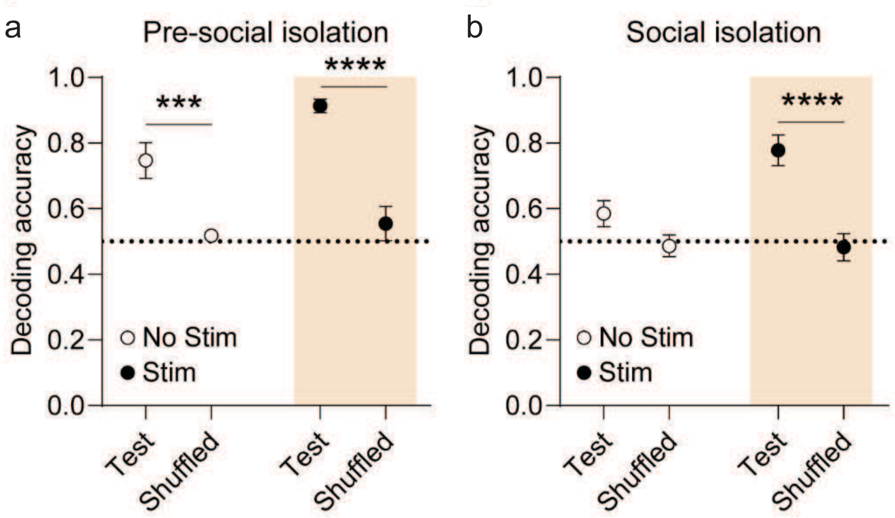

Supplementary Figure 8. BLA-mPFC terminal stimulation increases mPFC decoding of social interaction.

a, mPFC decodes social interaction compared to chance levels under non-stimulated and BLA-mPFC stimulated conditions (two-way ANOVA, main effect of group: F (1, 36) = 54.29, *****p* < 0.0001, main effect of stimulation: F (1, 36) = 6.47, **p* = 0.01, Sidak's post hoc test, ****p* < 0.001, *****p* < 0.0001). **b,** Social isolation abolishes mPFC decoding of social isolation, which is rescued by BLA-mPFC stimulation (two-way ANOVA, interaction effect: F (1, 36) = 5.95, **p* < 0.05, main effect of group: F (1, 36) = 23.78, *****p* < 0.0001, main effect of stimulation: F (1, 36) = 5.47, **p* = 0.01, Sidak's post hoc test, *****p* < 0.0001). Error bars indicate ±SEM.

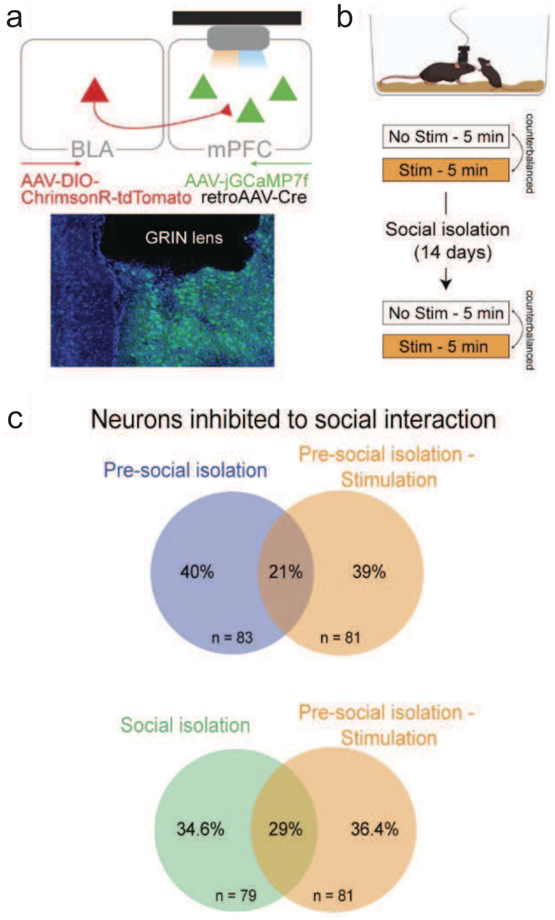

Supplementary Figure 9. BLA-mPFC terminal stimulation increases mPFC decoding of social interaction.

a, Viral strategy and endoscopic lens implant for simultaneous monitoring of mPFC activity and stimulation of BLA terminals in the mPFC. **b,** Calcium recordings during the resident-juvenile intruder task were performed before and during social isolation and BLA-mPFC stimulation was counterbalanced across two days. **c,** BLA-mPFC terminal stimulation recruited a similar proportion of the neurons excited to social interaction, as determined by Wilcoxon signed-rank test, during social isolation versus pre-social isolation conditions. Error bars indicate ±SEM.

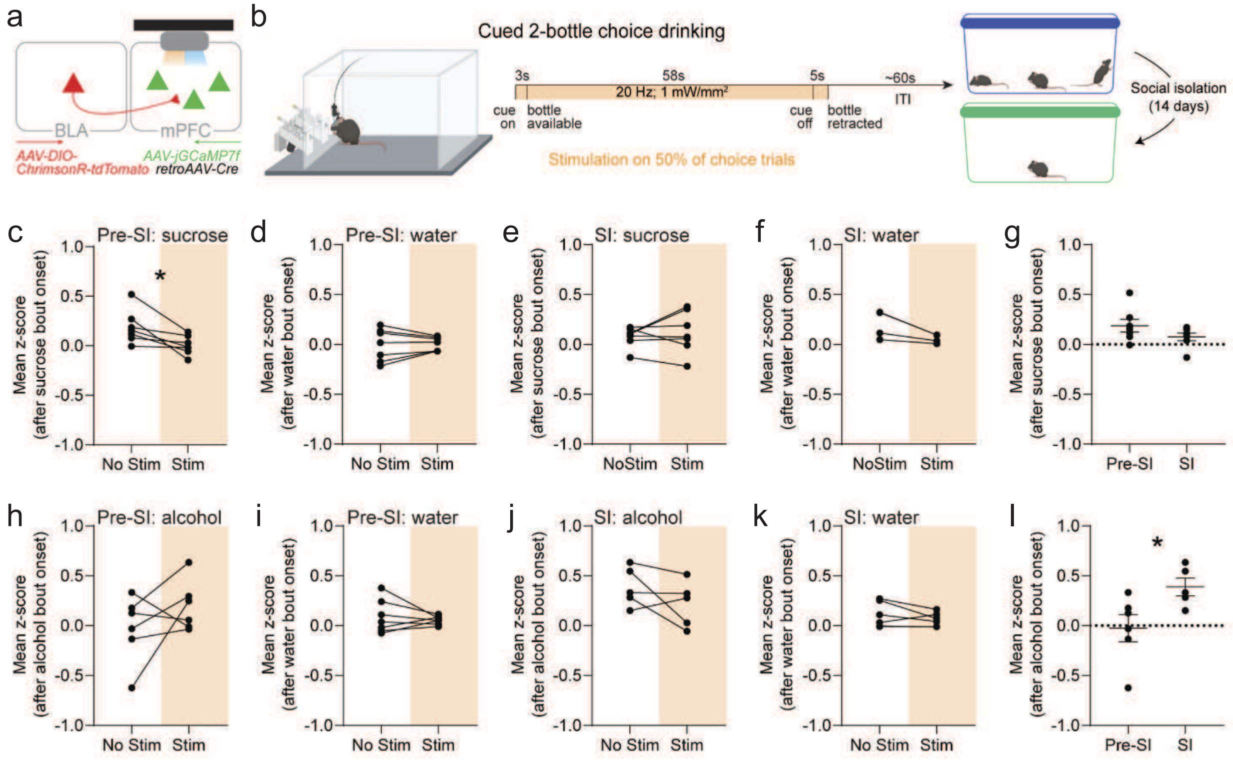

Supplementary Figure 10. Mean mPFC responses to sucrose, alcohol, and water under BLA-mPFC non-stimulated and stimulated conditions and before and during social isolation for individual mice.

a, Viral strategy and endoscopic lens implant for simultaneous monitoring of mPFC activity and stimulation of BLA terminals in the mPFC. **b,** To measure the impact of BLA stimulation on mPFC dynamics during drinking, we used the cued-two-bottle choice drinking task, wherein BLA-mPFC stimulation occurred during 50% of trials, which was repeated pre- and during social isolation. **c,d,** BLA-mPFC stimulation significantly decreased the mean mPFC response to sucrose (c; paired t-test, *$p < 0.05$) with no effect on mPFC responses to water (d) under pre-social isolation conditions. **e,f,** BLA-mPFC stimulation did not alter mPFC responses to sucrose (e) or water (f) during social isolation. **g,** Mean mPFC responses to sucrose before and during social isolation was not significantly different. **h,i,** BLA-mPFC stimulation did not alter mPFC responses to alcohol (h) or water (i) pre-social isolation. **j,k,** BLA-mPFC stimulation did not alter mPFC responses to alcohol (j) or water (k) during social isolation. **l,** Social isolation significantly increased the mean mPFC responses to alcohol (unpaired t-test, *$p < 0.05$). Error bars indicate ±SEM.

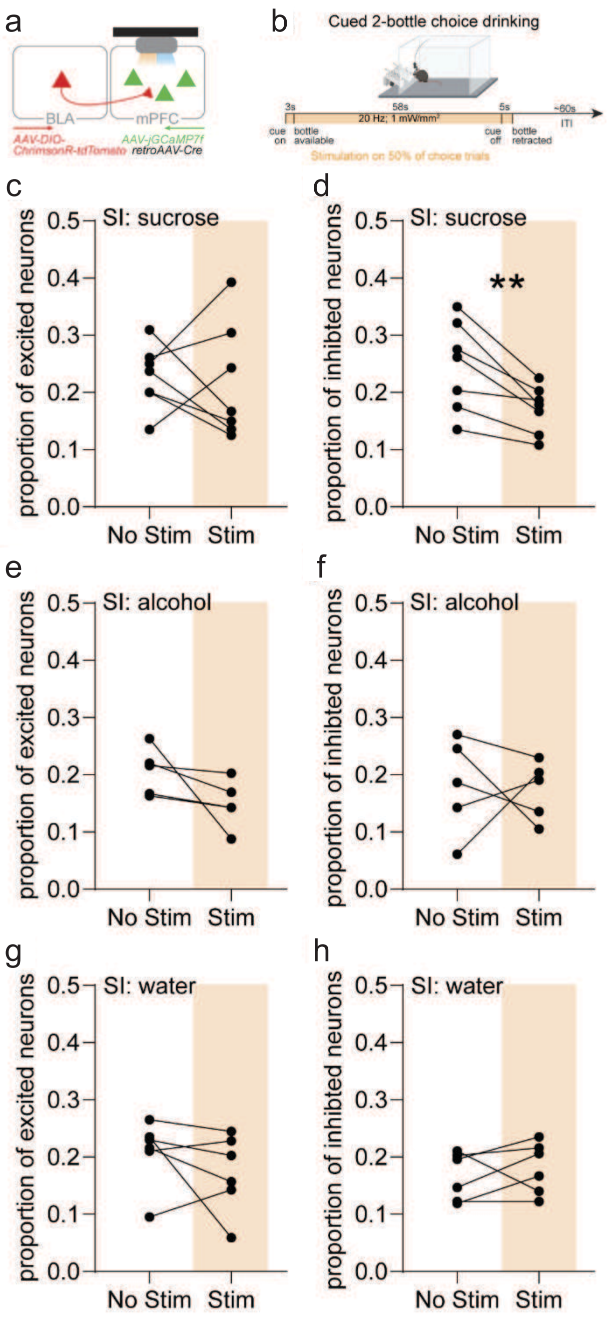

Supplementary Figure 11. Proportion of mPFC responsive neurons to sucrose, alcohol, and water during social isolation under BLA-mPFC stimulated and non-stimulated conditions.

a, Viral strategy and endoscopic lens implant for simultaneous monitoring of mPFC activity and stimulation of BLA terminals in the mPFC. **b,** To measure the impact of BLA stimulation on mPFC dynamics during drinking, we used the cued-two-bottle choice drinking task, wherein BLA-mPFC stimulation occurred during 50% of trials, during social isolation. **c,d,** BLA-mPFC stimulation significantly decreased the proportion of

mPFC neurons inhibited to sucrose (d; paired t-test, $^{**}p < 0.01$) with no effect on mPFC neurons excited to sucrose (c), based on a mean Z-score response ±1.98, during social isolation. **e,f,** BLA-mPFC stimulation did not alter the proportion of mPFC neurons responsive to alcohol during social isolation. **g,h,** BLA-mPFC stimulation did not alter the proportion of mPFC neurons responsive to water during social isolation. Error bars indicate ±SEM.

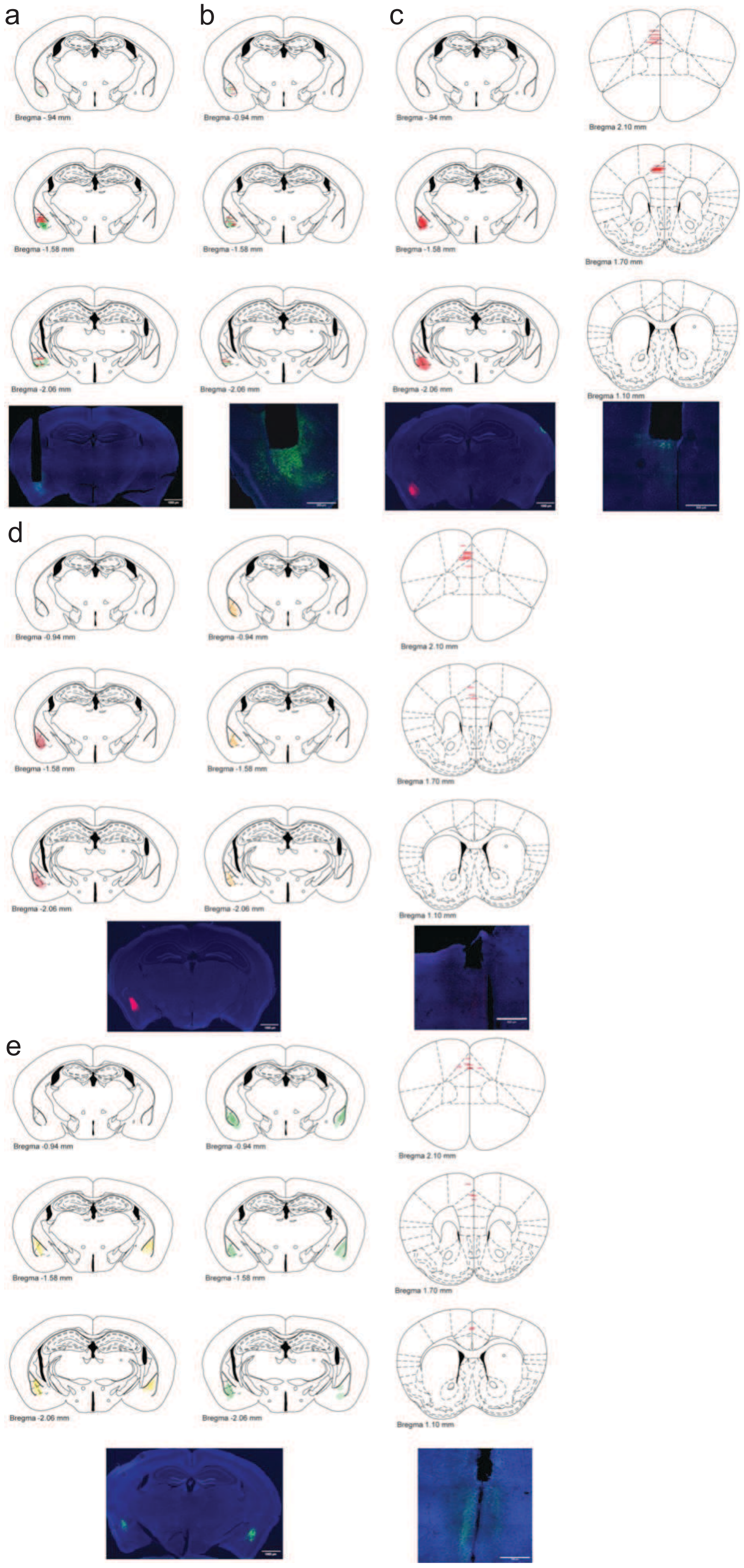
a
b
c
d
e

Supplementary Figure 12. Histological verification of injection sites and implants.

a,b, Injection sites for GCaMP7f and GRIN lens implant locations for non-specific BLA and BLA-mPFC calcium recordings, respectively. **c,** ChrimsonR expression in the BLA and GRIN lens implant locations in mPFC for simultaneous excitation of BLA-mPFC terminals and mPFC calcium recordings. **d,** ChrimsonR or tdTomato expression in BLA and fiber optic implant locations in mPFC for excitation of BLA-mPFC terminals during social interaction. **e,** NpHR or eYFP expression in the BLA and fiber optic implant locations in the mPFC for inhibition of BLA-mPFC terminals during social interaction and cued-2BC.

		Resting membrane potential (mV)	Capacitance (pF)	Input resistance (mΩ)	Threshold (pA)
Pre-social isolation	Non-specific BLA	-60.95 ± 3.9	156.1 ± 6.0	112.2 ± 6.1	164.6 ± 11.9
	BLA-mPFC	-68.4 ± 1.0	157.8 ± 8.0	98.6 ± 5.8	181.9 ± 12.7
Social isolation	Non-specific BLA	-68.9 ± 0.8*	183.0 ± 8.3*	111.8 ± 5.1	154.5 ± 9.4
	BLA-mPFC	-68.4 ± 1.2	163.9 ± 8.1	119.7 ± 7.3*	145.2 ± 13.9

Supplementary Table 1. Electrophysiological properties of non-specific BLA and BLA-mPFC neurons before and during social isolation. Unpaired t-test between pre-social isolation and social isolation conditions for each population, *$p < 0.05$.

BLA-mPFC:		Resting membrane potential (mV)	Capacitance (pF)	Input resistance (mΩ)	Threshold (pA)
Pre-social isolation	Rank 1	-65.6 ± 2.0	184.5 ± 19.8	74.0 ± 6.4	240.0 ± 30.3
	Rank 2	-67.5 ± 3.5	157.9 ± 14.8	122.8 ± 8.2	145.0 ± 9.6
	Rank 3	-69.8 ± 1.2	100.8 ± 7.5	146.6 ± 9.3	170.0 ± 13.6
Social isolation	Rank 1	-69.6 ± 2.0	167.6 ± 9.8	129.7 ± 10.8**	123.6 ± 22.9*
	Rank 2	-66.2 ± 4.3	156.9 ± 15.3	101.5 ± 12.4	174.0 ± 26.3
	Rank 3	-68.7 ± 2.0	127.0 ± 14.2	146.9 ± 18.7	140.0 ± 22.7

Supplementary Table 2. Electrophysiological properties of BLA-mPFC neurons from each social rank before and during social isolation. Unpaired t-test between pre-social isolation and social isolation conditions for each social rank, *$p < 0.05$, **$p < 0.01$

Non-specific BLA:		Resting membrane potential (mV)	Capacitance (pF)	Input resistance (mΩ)	Threshold (pA)
Pre-social isolation	Rank 1	-70.9 ± 1.7	153.2 ± 8.5	113.0 ± 11.8	174.0 ± 24.6
	Rank 2	-63.2 ± 2.9	150.2 ± 15.1	124.2 ± 10.3	120.0 ± 17.3
	Rank 3	-66.7 ± 1.4	153.8 ± 9.6	105.7 ± 8.8	175.6 ± 16.2
Social isolation	Rank 1	-69.4 ± 0.9	179.6 ± 11.7	113.5 ± 7.1	148.8 ± 13.1
	Rank 2	-70.2 ± 1.3	194.5 ± 19.6	115.8 ± 10.0	165.7 ± 17.6
	Rank 3	-69.3 ± 1.5	177.4 ± 15.5	99.7 ± 7.9	148.0 ± 20.5

Supplementary Table 3. Electrophysiological properties of non-specific BLA neurons from each social rank before and during social isolation.

Figure panel	Number of mice	Age	Histology
**1a-m**	16	8-10 weeks	n/a
**2a-k**	28	8-10 weeks	Supp Figure 12a,b
**2l-r**	18	8-10 weeks	Supp Figure 5e
**3c-d**	16	8-10 weeks	Supp Figure 12d
**3e-f**	16	8-10 weeks	Supp Figure 12e
**3g-s**	6	8-10 weeks	Supp Figure 12c
**4a-q**	9	8-10 weeks	Supp Figure 12c
**4r-u**	16	8-10 weeks	Supp Figure 12e

Supplementary Table 4. Age and number of mice used in main text experiments.
